# Supplementary material for: Survey of metaproteomics software tools for functional microbiome analysis
Source: PLoS One. 2020 Nov 10;15(11):e0241503. doi: 10.1371/journal.pone.0241503 (PMC7654790; doi:10.1371/journal.pone.0241503)
Supplement: S1 File — (DOCX) [file pone.0241503.s001.docx]

**SUPPLEMENTARY SECTION**

**Supplement S1**

**Supplement S1 Table 1: All ontologies**

| **Tool** | **EggNOG** | **MEGAN** | **MetaGOmics** | **MPA** | **Prophane** | **Unipept** |
| --- | --- | --- | --- | --- | --- | --- |
| **Type of functional annotation** | Proteins | eggNOG orthologous groups | GO terms | Proteins | Protein families, eggNOG orthologous groups | GO terms |
| **Total number of annotation groups** | 18,440 | 1,665 | 2,829 | 23,169 | 3,999 | 3,471 |
| **Total (and unique) number of translated all GO terms** | 533,066 (6,155) | 76,529 (4,155) | 2,829 (2,829) | 77,204 (1,056) | 189,054 (2,598) | 3,471 (3,471) |
| **Total number of all GO terms exclusive to the tool** | 2,612 | 401 | 485 | 8 | 50 | 901 |
| **Total number of all expanded GO terms** | 6,443 | 4,367 | 3,151 | 2,133 | 2,767 | 6,105 |
| **Total number of all expanded GO terms exclusive to the tool** | 2,043 | 262 | 325 | 4 | 39 | 1,315 |

Peptide search results from the oral dysbiosis dataset pair (see methods) were processed to provide appropriate inputs for functional analysis. The outputs from the data processing through these software tools shows that the total number of functional terms differed for each software tool. To facilitate a comparison, the functional terms were converted into GO terms. The number of GO terms for all ontologies ranged from as low as 1056 (for MetaProteomeAnalyzer) to 6155 (for EggNOG mapper). For a more even comparison, we expanded the GO terms using the metaQuantome tool. For all GO terms, the number of expanded terms ranged from 2,133 (for MPA) to 6,443 (EggNOG mapper). Notably, eggNOG-mapper identified the largest number of expanded GO terms (2,612) and expanded GO terms (2,043).

**Supplement S1 Table 2: Biological Process**

|  | **EggNOG** | **MEGAN** | **MetaGOmics** | **MPA** | **Prophane** | **Unipept** |
| --- | --- | --- | --- | --- | --- | --- |
| **Type of functional annotation** | Proteins | eggNOG orthologous groups | GO terms | Proteins | Protein families, eggNOG orthologous groups | GO terms |
| **Total number of annotation groups** | 18,440 | 1,665 | 2,829 | 23,169 | 3,999 | 3,471 |
| **Total (and unique) number of translated GO terms for all ontologies** | 533,066 (6,155) | 76,529 (4,155) | 2,829 (2,829) | 77,204 (1,056) | 189,054 (2,598) | 3,471 (3,471) |
| **Total (and unique) number of translated biological process GO terms** | 319,904 (3,964) | 44,771 (2,130) | 1,685 (1,685) | 21,271 (364) | 107,046 (1,316) | 1,338 (1,338) |
| **Total number of biological process GO terms exclusive to the tool** | 1,981 | 191 | 305 | 6 | 21 | 359 |
| **Total number of biological process expanded GO terms** | 4,310 | 2,332 | 1,914 | 1,063 | 1,447 | 3,281 |
| **Total number of biological process expanded GO terms exclusive to the tool** | 1,595 | 119 | 212 | 3 | 21 | 701 |

Peptide search results from the oral dysbiosis dataset pair (see methods) were processed to provide appropriate inputs for functional analysis. To facilitate comparison, the functional terms were collapsed into GO terms and were filtered to GO terms within the biological process sub-ontology. The number of GO terms for biological process ontology ranged from as low as 364 (for MetaProteomeAnalyzer) to 3,964 (for EggNOG mapper). The results from MEGAN and MetaGOmics also showed a high number of biological process GO terms, with eggNOG-mapper exclusively identifying most number of biological process GO terms (2,149). Expanded versions of the biological process GO terms ranged from as less as 1,063 (for MPA) to 4,310 (eggNOG-mapper). Notably, eggNOG-mapper exclusively identified the most exclusive expanded biological process GO terms (1,595) and MPA had the fewest exclusive expanded biological process GO terms (3).

**Supplement S1 Table 3: Cellular component**

|  | **EggNOG** | **MEGAN** | **MetaGOmics** | **MPA** | **Prophane** | **Unipept** |
| --- | --- | --- | --- | --- | --- | --- |
| **Type of functional annotation** | Proteins | eggNOG orthologous groups | GO terms | Proteins | Protein families, eggNOG orthologous groups | GO terms |
| **Total number of annotation groups** | 18,440 | 1,665 | 2,829 | 23,169 | 3,999 | 3,471 |
| **Total (and unique) number of translated GO terms for all ontologies** | 533,066 (6,155) | 76,529 (4,155) | 2,829 (2,829) | 77,204 (1,056) | 189,054 (2,598) | 3,471 (3,471) |
| **Total (and unique) number of translated cellular component GO terms** | 106,223 (640) | 7,118 (314) | 241 (241) | 13,809 (56) | 17,854 (164) | 389 (389) |
| **Total number of cellular component GO terms exclusive to the tool** | 310 | 31 | 64 | 0 | 6 | 145 |
| **Total number of cellular component expanded GO terms** | 667 | 342 | 235 | 96 | 185 | 575 |
| **Total number of cellular component expanded GO terms exclusive to the tool** | 244 | 25 | 33 | 0 | 7 | 167 |

Peptide search results from the oral dysbiosis dataset pair (see methods) were processed to provide appropriate inputs for functional analysis for GO terms within the cellular component sub-ontology. The number of GO terms for the cellular component sub-ontology ranged from as low as 56 (for MetaProteomeAnalyzer) to 640 (for eggNOG-mapper). MEGAN and MetaGOmics also showed a high number of cellular component GO terms, with eggNOG-mapper identifying the largest number of cellular component GO terms (310) exclusive to the tool. The expanded set of cellular component GO terms ranged from as few as 96 (for MPA) to 667 (eggNOG-mapper). Notably, eggNOG-mapper identified the largest number of exclusive expanded GO terms (244) and MPA had no expanded GO terms exclusive to the tool.

**Supplement S2**


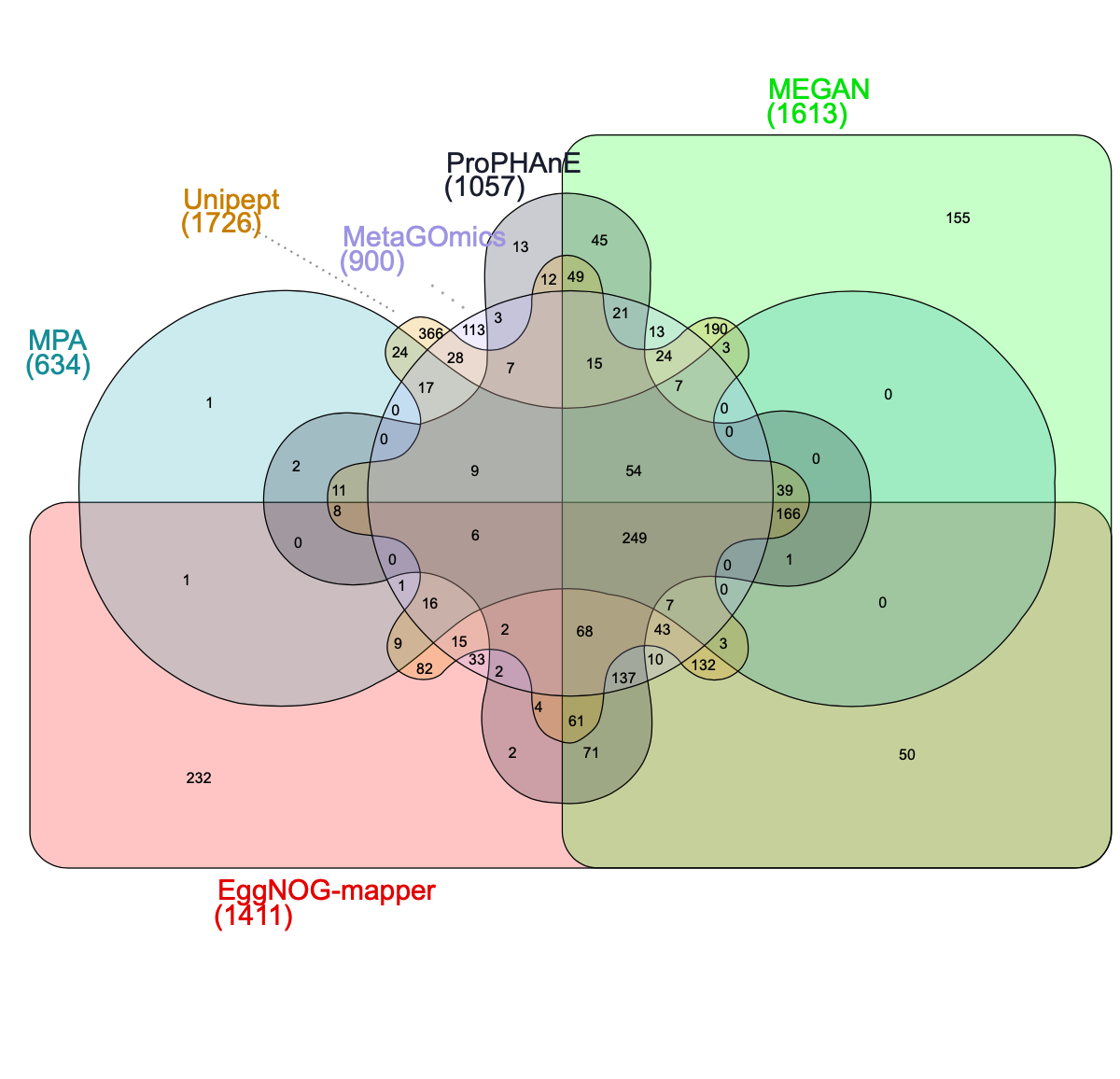
 **Supplement S2 Figure 1A.** An Edwards-Venn diagram that illustrates all intersection combinations between the unique GO term sets (molecular function) of all six functional tools. For example, 249 unique terms were found to be common throughout all six tools, EggNOG-mapper had the most exclusive terms (232), and EggNOG-mapper and MEGAN had 50 terms in common.


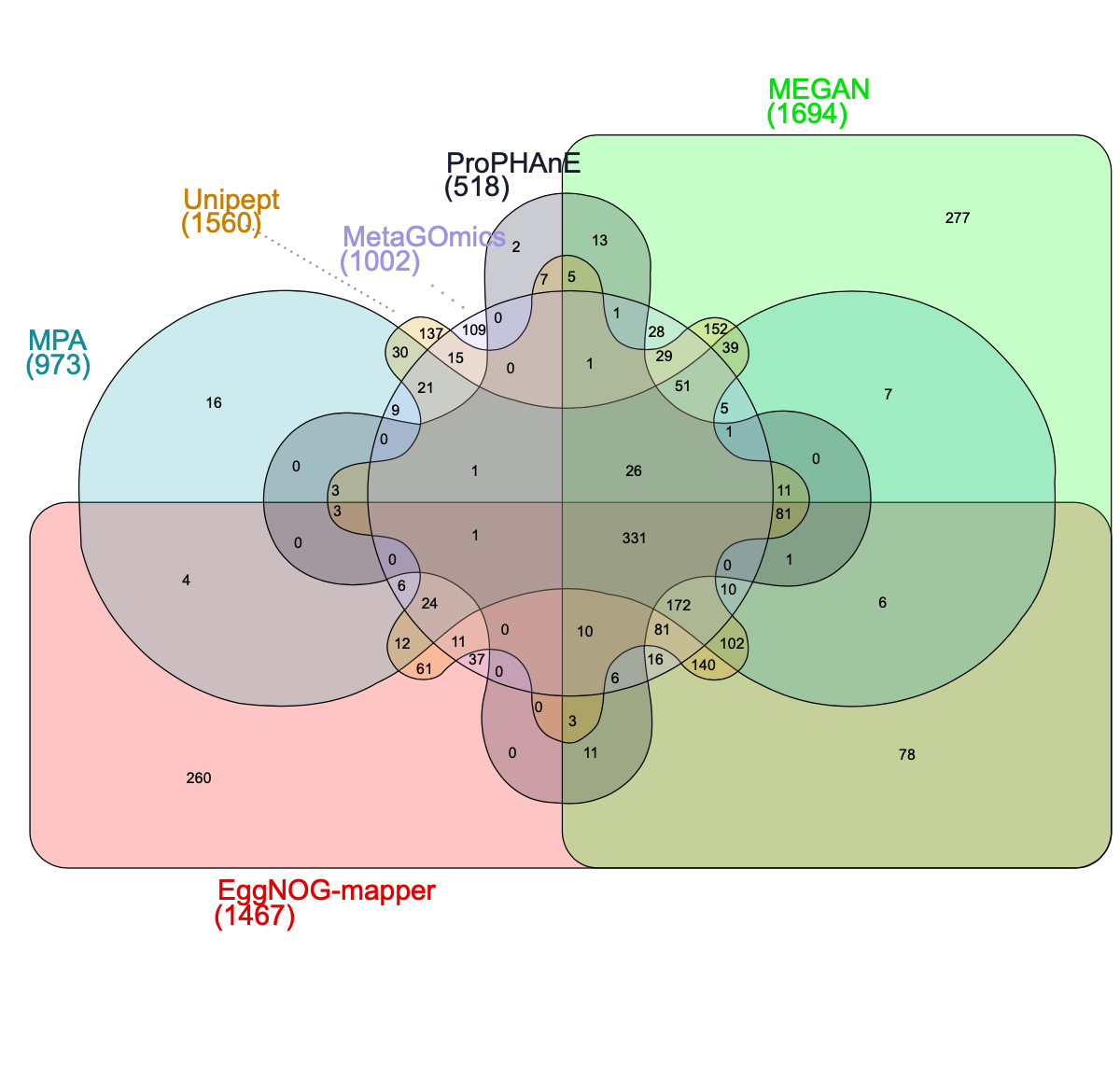


**Supplement S2 Figure 1B.** An Edwards-Venn diagram that illustrates all intersection combinations between the expanded unique GO term sets (molecular function) of all six functional tools. For example, 331 unique terms were found to be common throughout all six tools, MEGAN had the most exclusive terms (277), and no terms were found in common between MetaGOmics, ProPHAnE, and Unipept.

**
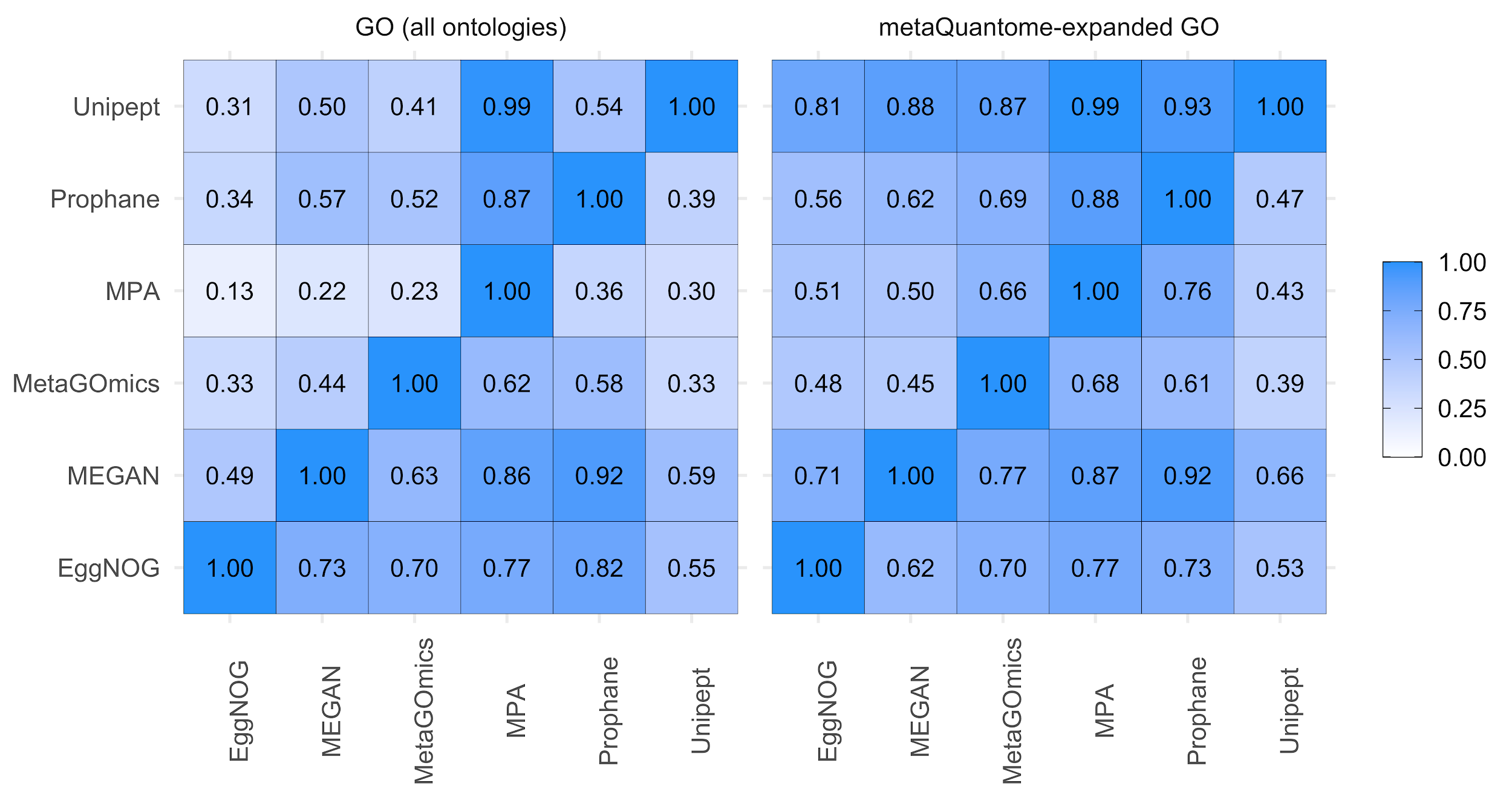
**

**Supplement 2 Figure 2: Qualitative comparison of functional tools (All ontologies)**

A) The overlap of unique GO terms (left) and expanded GO terms (right) was compared amongst the six functional tools. Values were calculated as a fraction of the size of term intersection (between the tools labeled on the column and row) over the total term size of the tool listed on the horizontal axis (column). Each functional analysis software tool was compared against each other.

Pairwise overlap analysis was performed on unique translated GO terms from all ontologies (sizes indicated in **S1 Table 1**). The tool pairings that had the most overlap were Unipept in MPA (99% of MPA’s 1,056 terms were found in Unipept’s 3,471 terms) and MEGAN in ProPHAnE (92% of ProPHAnE’s 2,598 terms were found in MEGAN’s 4,155). Generally, tools with larger GO sets (e.g., eggNOG-mapper at 6,155) had better coverage in other tools’ GO sets while smaller GO sets (e.g., ProPHAnE at 1,267) did not. In the metaQuantome-expanded versions of these GO sets, there is an overall improvement, notably with tools that had the most expansion such as Unipept. Relative to GO term comparison, expanded GO term comparison shows an improvement due to an expanded representation of each term.


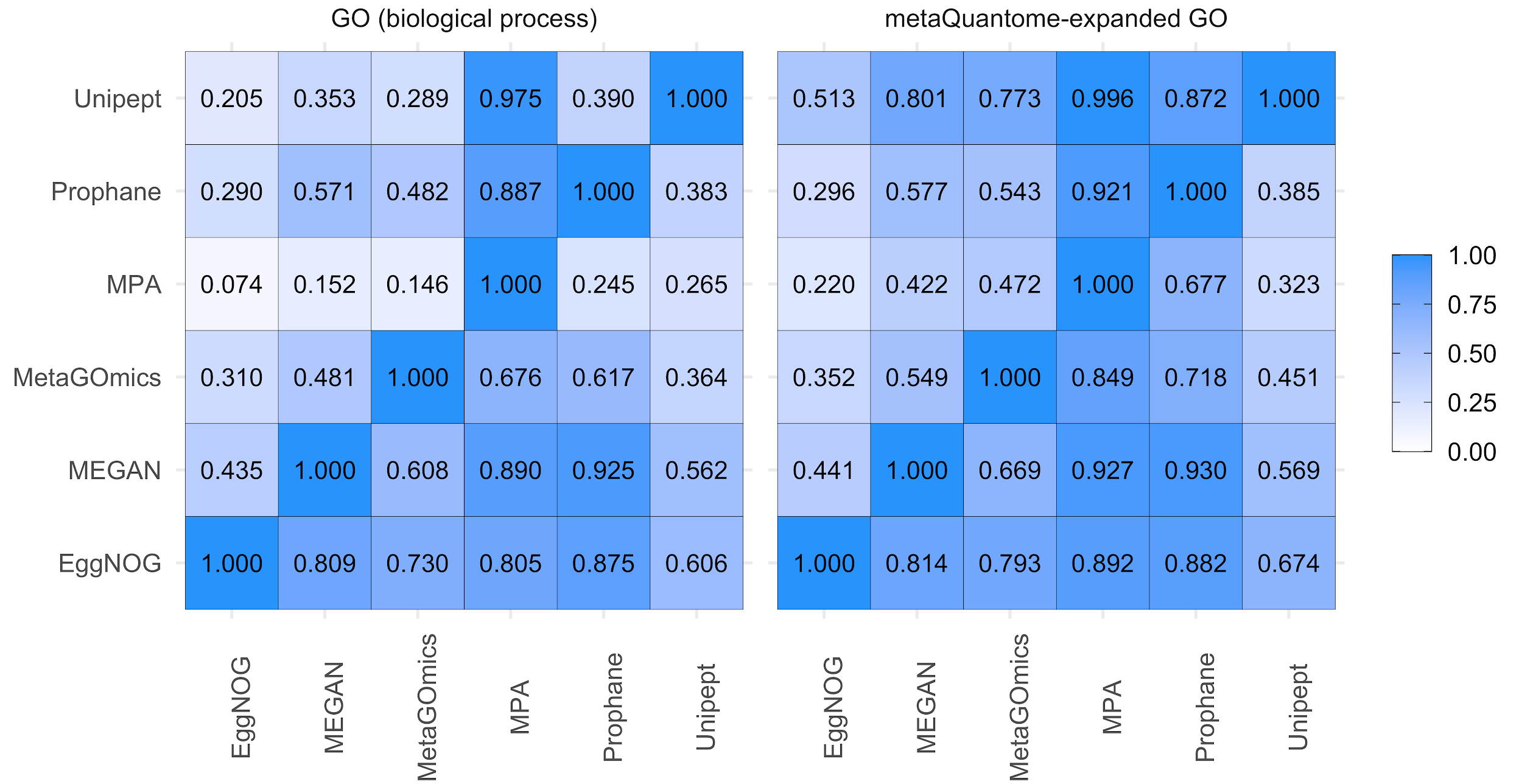


**Supplement 2 Figure 3: Qualitative comparison of functional tools (Biological process)**

A) The overlap of unique GO terms (left) and expanded GO terms (right) was compared amongst the six functional tools. Values were calculated as a fraction of the size of term intersection (between the tools labeled on the column and row) over the total term size of the tool listed on the horizontal axis (column). Each functional analysis software tool was compared against each other. .

Pairwise overlap analysis was performed on unique translated GO terms from biological process ontologies (sizes indicated in **S1 Table 2**). The tool pairings that had the most overlap were Unipept in MPA (98% of MPA’s 364 terms were found in Unipept’s 1,338 terms) and MEGAN in ProPHAnE (93% of ProPHAnE’s 1,316 terms were found in MEGAN’s 2,130). Generally, tools with larger GO sets (e.g., eggNOG-mapper at 3,964) had better coverage in other tools’ GO sets while smaller GO sets (e.g., MPA at 364) did not. In the metaQuantome-expanded versions of these GO sets, there is an overall improvement, notably with tools that had the most expansion (MPA and Unipept). Relative to GO term comparison, expanded GO term comparison shows an improvement due to an expanded representation of each term.


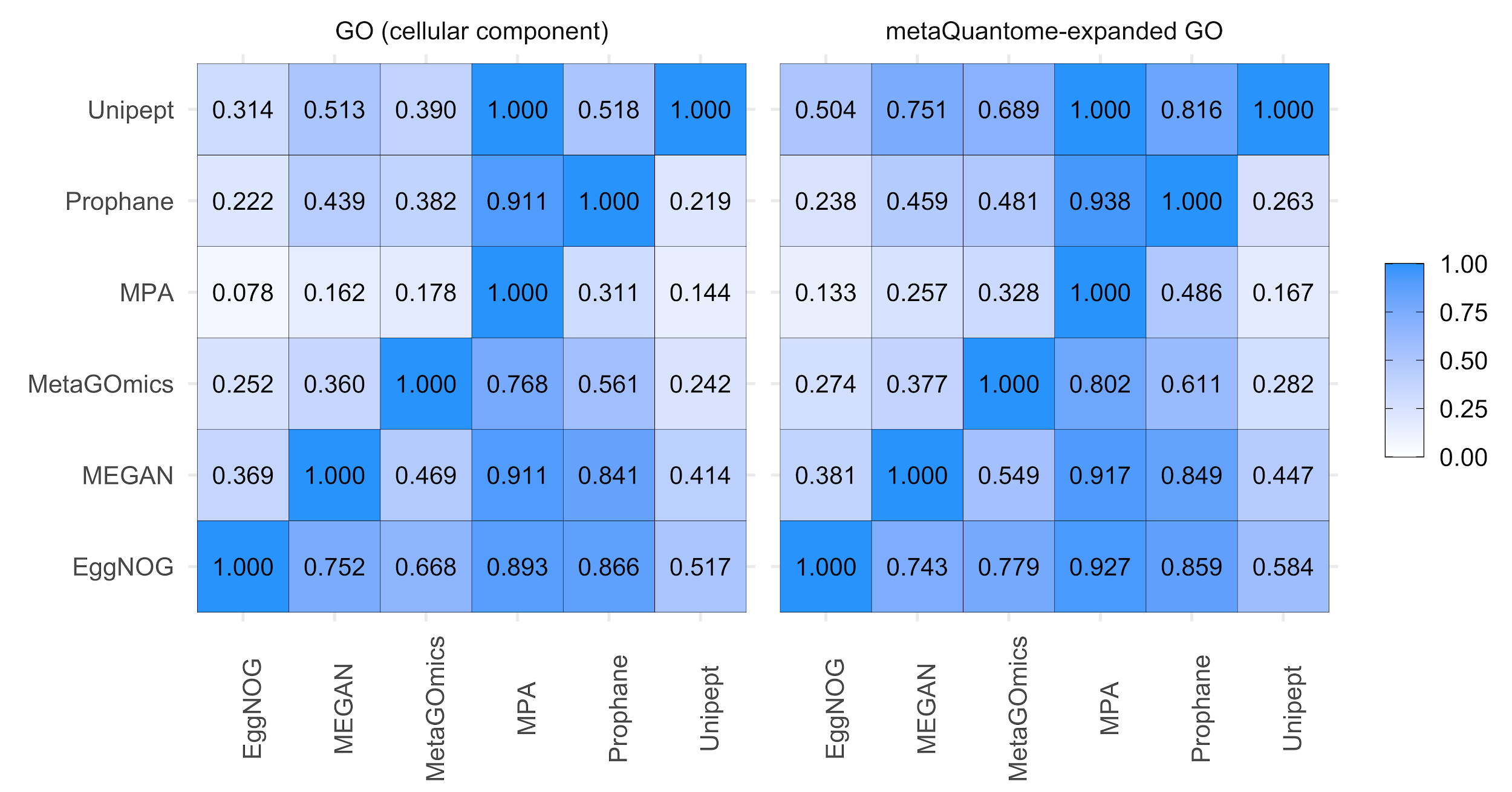


**Supplement 2 Figure 4: Qualitative comparison of functional tools (Cellular component)**

A) The overlap of unique GO terms (left) and expanded GO terms (right) was compared amongst the six functional tools. Values were calculated as a fraction of the size of term intersection (between the tools labeled on the column and row) over the total term size of the tool listed on the horizontal axis (column). Each functional analysis software tool was compared against each other. .

Pairwise overlap analysis was performed on unique translated GO terms from cellular component ontologies (sizes indicated in **S1 Table 3**). The tool pairings that had the most overlap were ProPHAnE in MPA (91% of MPA’s 56 terms were found in ProPHAnE’s 164 terms) and MEGAN in MPA (91% of MPA’s 56 terms were found in MEGAN’s 314). Generally, tools with larger GO sets (e.g., eggNOG-mapper at 640) had better coverage in other tools’ GO sets while smaller GO sets (e.g., MPA at 56) did not. In the metaQuantome-expanded versions of these GO sets, there is an overall improvement, notably with tools that had the most expansion (Unipept). Relative to GO term comparison, expanded GO term comparison shows a slight improvement due to an expanded representation of each term.

**Supplement S3**

**
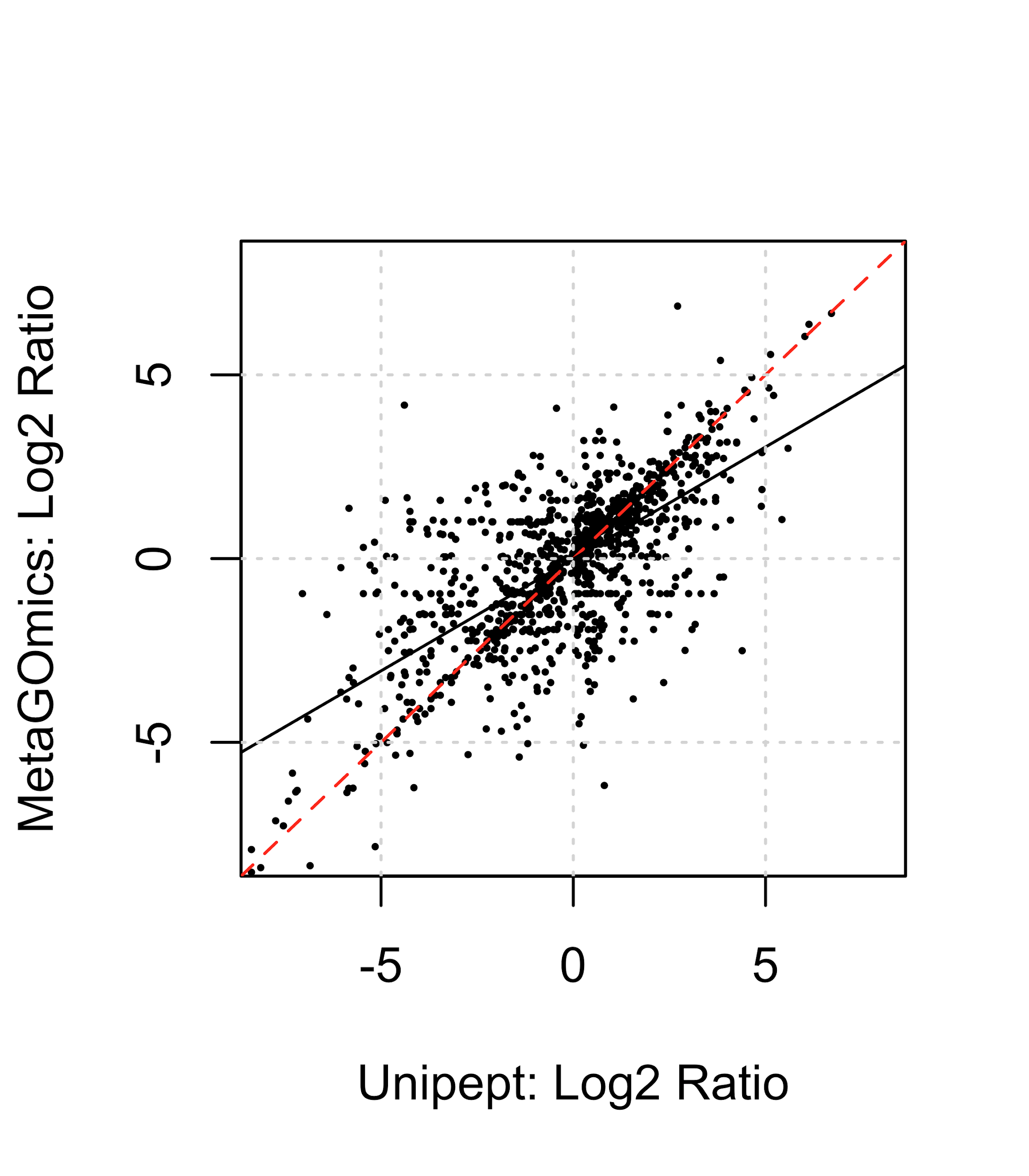
**

**Supplement S3 Figure 1:** Comparison of quantitative expression for all GO terms from Unipept and MetaGOmics. log2ratio of spectral counts ‘with sugar sample’ (WS) against ‘no sugar sample’ (NS) was calculated for metaGOmics and Unipept generated all GO terms. Unipept identified 6,105 GO terms (all ontologies), while MetaGOmics identified 3,151 GO terms (all ontologies). The data points in the figure represent quantitative values for 2,514 GO terms that overlapped between Unipept and metaGOmics.

Comparison of quantitative expression using spectral counts for all GO terms from Unipept and MetaGOmics was performed after normalization of spectral counts. Quantitative values of the overlapping GO terms were represented (Supplement S3 Fig 1). The Pearson coefficient of this quantitative comparison was found to be 0.680 with a significant P-value (<2.2e-16). Given that this is a quantitative comparison of the same dataset, a better quantitative correlation for overlapping all GO terms was expected amongst two functional tools which used the same annotation database (UniProtKB).


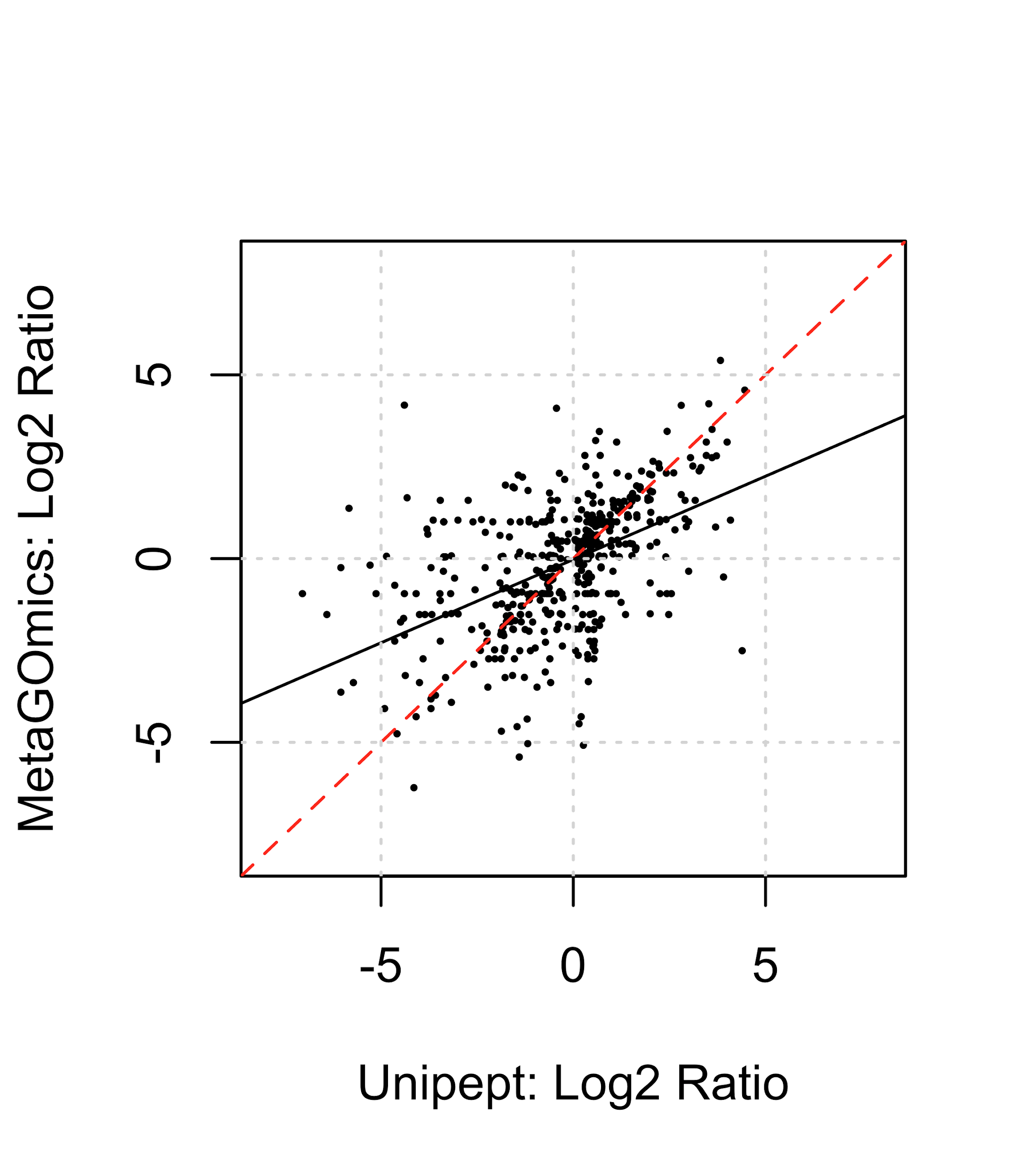


**Supplement S3 Figure 2:** Comparison of quantitative expression for biological process GO terms from Unipept and MetaGOmics. log2ratio of spectral counts ‘with sugar sample’ (WS) against ‘no sugar sample’ (NS) was calculated for metaGOmics and Unipept generated biological process GO terms. Unipept identified 1,338 biological process GO terms, while MetaGOmics identified 1,685 biological process GO terms. The data points in the figure represent quantitative values for 487 biological process GO terms that overlapped between Unipept and metaGOmics.

Comparison of quantitative expression using spectral counts for biological process GO terms from Unipept and MetaGOmics was performed after normalization of spectral counts. Quantitative values of the overlapping biological process GO terms were represented (Supplement S3 Fig 2). The Pearson coefficient of this quantitative comparison was found to be 0.506 with a significant P-value (< 2.2e-16). Given that this is a quantitative comparison of the same dataset, a better quantitative correlation for overlapping biological process GO terms was expected amongst two functional tools which used the same annotation database (UniProtKB).


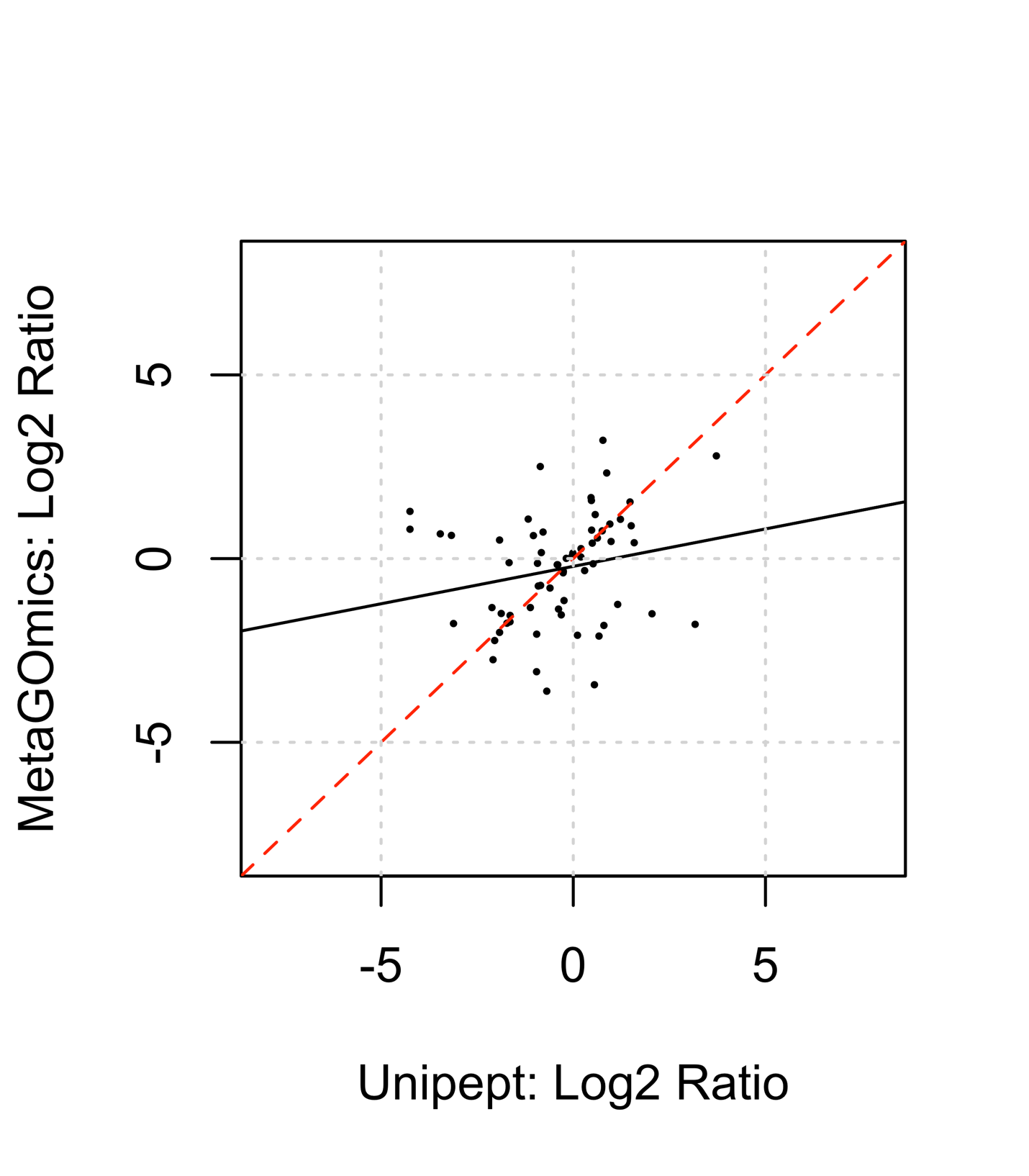


**Supplement S3 Figure 3:** Comparison of quantitative expression for cellular component GO terms from Unipept and MetaGOmics. log2ratio of spectral counts ‘with sugar sample’ (WS) against ‘no sugar sample’ (NS) was calculated for metaGOmics and Unipept generated cellular component GO terms. Unipept identified 389 cellular component GO terms, while MetaGOmics identified 241 cellular component GO terms. The data points in figure represent quantitative values for 94 cellular component GO terms that overlapped between Unipept and metaGOmics.

Comparison of quantitative expression using spectral counts for cellular component GO terms from Unipept and MetaGOmics was performed after normalization of spectral counts. Quantitative values of the overlapping cellular component GO terms were represented (Supplement S3 Fig 3). The Pearson coefficient of this quantitative comparison was found to be 0.239 with a significant P-value. Given that this is a quantitative comparison of the same dataset, a better quantitative correlation for overlapping cellular component GO terms was expected amongst two functional tools which used the same annotation database (UniProtKB).

**Supplement S4**

**Supplement S4 Table 1: Unipept (Expanded)**

| **GO Term** | **Fold Change** | **Unipept** | **EggNOG** | **MEGAN** | **MetaGOmics** | **MPA** | **Prophane** |
| --- | --- | --- | --- | --- | --- | --- | --- |
| glucosyltransferase activity | FC (WS / NS) | 1.32 (146 / 58.07) | -0.34 (39 / 49.64) | 0.21 (139 / 120) | 2.71 (117 / 17) | -0.03 (158.13 / 162) | 0.47 (0.94 / 0.4) |
|  | Percentile (%) | 10.008 | 56.34 | 44.332 | 6.569 | 43.741 | 34.369 |
| dextransucrase activity | FC (WS / NS) | 5.67 (101 / 1) | - | - | 5.71 (104 / 1) | 2.01 (7.03 / 1) | - |
|  | Percentile (%) | 0.016 | - | - | 0.095 | 11.158 | - |
| pyruvate oxidase activity | FC (WS / NS) | 5.38 (82 / 1) | - | - | 5.13 (69 / 1) | - | 0.82 (1 / 0.13) |
|  | Percentile (%) | 0.049 | - | - | 0.222 | - | 15.938 |
| glyceraldehyde-3-phosphate dehydrogenase (NADP+) (non-phosphorylating) activity | FC (WS / NS) | 5.04 (65 / 1) | - | - | 5.02 (64 / 1) | - | - |
|  | Percentile (%) | 0.066 | - | - | 0.254 | - | - |
| fructuronate reductase activity | FC (WS / NS) | 4.7 (51 / 1) | - | 0 (1 / 1) | - | - | - |
|  | Percentile (%) | 0.098 | - | 56.698 | - | - | - |
| CoA-transferase activity | FC (WS / NS) | -5.81 (25 / 1459.16) | -0.67 (518 / 824.17) | -6.6 (70 / 6900) | -5.24 (29 / 1134) | -14.26 (1 / 39110) | -3.2 (1 / 17.4) |
|  | Percentile (%) | 99.705 | 65.963 | 99.657 | 98.096 | 100 | 99.53 |
| acetyl-CoA C-acyltransferase activity | FC (WS / NS) | -8.26 (1 / 610.64) | -8.47 (1 / 709.91) | -8.59 (1 / 768) | -8.37 (1 / 660) | -12.33 (1 / 10328) | -2.98 (1 / 14.8) |
|  | Percentile (%) | 100 | 99.984 | 99.84 | 99.905 | 99.812 | 99.422 |
| butyrate-acetoacetate CoA-transferase activity | FC (WS / NS) | -7.36 (1 / 326.86) | - | - | -5.85 (1 / 114) | -5.69 (1 / 102) | 0.26 (1 / 0.67) |
|  | Percentile (%) | 99.951 | - | - | 98.667 | 89.78 | 44.922 |
| glutaconate CoA-transferase activity | FC (WS / NS) | -7.41 (1 / 339.03) | - | - | -7.13 (1 / 279) | -6.85 (1 / 230) | 0 (1 / 1) |
|  | Percentile (%) | 99.967 | - | - | 99.778 | 92.405 | 57.752 |
| acetyl-CoA C-acetyltransferase activity | FC (WS / NS) | -7.54 (1 / 369.94) | -7.48 (1 / 354.96) | -7.59 (1 / 384) | -7.37 (1 / 330) | -12.32 (1 / 10248) | -2.44 (1 / 9.87) |
|  | Percentile (%) | 99.984 | 99.829 | 99.817 | 99.81 | 99.766 | 99.06 |

**Supplement S4 Table 1.** Comparison of the top five upregulated and top five downregulated expanded molecular function GO terms of Unipept with the molecular function GO terms from the other tools from the oral dysbiosis dataset. Fold changes are featured here (descending for Unipept). For other tools, if there are multiple GO terms that match the top term, then the term with the highest absolute fold change is displayed. Additionally, spectral counts are indicated for “with sucrose” and “no sucrose” (WS / NS) conditions which are used to calculate the displayed fold change (FC) = log_2_($\frac{WS + 1}{NS + 1}$). Percentiles are included to indicate the position of that particular term in that GO set containing all ontologies and expanded GO terms (0 = most upregulated; 100 = most downregulated).

When expanded, some Gene Ontology terms like ‘glucosyltransferase activity’ (topmost term from the unexpanded Table 2 in the main text) had lower fold changes as compared to the unexpanded Table 2. As a result of expansion, other Gene Ontology terms were introduced into the top five differentially expressed GO terms, such as ‘peptide deformylase activity’. Overall, most Gene Ontology terms that were expanded stay relatively unchanged, even amongst protein-level tools that have duplicate Gene Ontology terms.

**Supplement S4 Table 2:Unipept (Expanded + Filtered)**

| **GO Term** | **Fold Change** | **Unipept** | **EggNOG** | **MEGAN** | **MetaGOmics** | **MPA** | **Prophane** |
| --- | --- | --- | --- | --- | --- | --- | --- |
| glucosyltransferase activity | FC (WS / NS) | 1.32 (146 / 58.07) | -0.34 (39 / 49.64) | - | 2.71 (117 / 17) | -0.03 (158.13 / 162) | 0.47 (0.94 / 0.4) |
|  | Percentile (%) | 10.297 | 59.68 | - | 5.801 | 46.372 | 34.064 |
| dextransucrase activity | FC (WS / NS) | 5.67 (101 / 1) | - | - | 5.71 (104 / 1) | 2.01 (7.03 / 1) | - |
|  | Percentile (%) | 0.025 | - | - | 0.049 | 10.087 | - |
| pyruvate oxidase activity | FC (WS / NS) | 5.38 (82 / 1) | - | - | 5.13 (69 / 1) | - | 0.82 (1 / 0.13) |
|  | Percentile (%) | 0.049 | - | - | 0.197 | - | 16.539 |
| glyceraldehyde-3-phosphate dehydrogenase (NADP+) (non-phosphorylating) activity | FC (WS / NS) | 5.04 (65 / 1) | - | - | 5.02 (64 / 1) | - | - |
|  | Percentile (%) | 0.074 | - | - | 0.246 | - | - |
| fructuronate reductase activity | FC (WS / NS) | 4.7 (51 / 1) | - | 0 (1 / 1) | - | - | - |
|  | Percentile (%) | 0.123 | - | 57.172 | - | - | - |
| CoA-transferase activity | FC (WS / NS) | -5.81 (25 / 1459.16) | - | -6.6 (70 / 6900) | - | -14.26 (1 / 39110) | -3.2 (1 / 17.4) |
|  | Percentile (%) | 99.73 | - | 99.699 | - | 100 | 99.726 |
| acetyl-CoA C-acyltransferase activity | FC (WS / NS) | - | - | - | - | - | - |
|  | Percentile (%) | - | - | - | - | - | - |
| butyrate-acetoacetate CoA-transferase activity | FC (WS / NS) | -7.36 (1 / 326.86) | - | - | -5.85 (1 / 114) | -5.69 (1 / 102) | 0.26 (1 / 0.67) |
|  | Percentile (%) | 99.951 | - | - | 98.869 | 90.784 | 45.455 |
| glutaconate CoA-transferase activity | FC (WS / NS) | -7.41 (1 / 339.03) | - | - | -7.13 (1 / 279) | -6.85 (1 / 230) | 0 (1 / 1) |
|  | Percentile (%) | 99.975 | - | - | 99.803 | 93.324 | 59.036 |
| acetyl-CoA C-acetyltransferase activity | FC (WS / NS) | -7.54 (1 / 369.94) | -7.48 (1 / 354.96) | -7.59 (1 / 384) | -7.37 (1 / 330) | -12.32 (1 / 10248) | -2.44 (1 / 9.87) |
|  | Percentile (%) | 100 | 99.833 | 99.866 | 99.853 | 99.71 | 99.343 |

**Supplement S4 Table 2.** Comparison of the top five upregulated and top five downregulated filtered molecular function GO terms of Unipept with the molecular function GO terms from the other tools from the oral dysbiosis dataset. Fold changes are featured here (descending for Unipept). For other tools, if there are multiple GO terms that match the top term, then the term with the highest absolute fold change is displayed. Additionally, spectral counts are indicated for “with sucrose” and “no sucrose” (WS / NS) conditions which are used to calculate the displayed fold change (FC) = log_2_($\frac{WS + 1}{NS + 1}$). Percentiles are included to indicate the position of that particular term in that GO set containing all ontologies but only filtered GO terms (0 = most upregulated; 100 = most downregulated).

The filtering step removes terms that do not have more than one child GO term supporting it from the expansion step. Majority of the terms were preserved after filtering, while only a few terms were filtered out. For example, GO terms such as such as ‘D-lysine, 5,6-aminomutase activity’, which is a leaf term (i.e., has no possible child GO term), and ‘oxidoreductase activity, acting on the aldehyde or oxo group of donors, oxygen as acceptor’ (which has quite a few possible children GO terms) were filtered out.

**Supplement S4 Table 3: EggNOG mapper**

|  | **Fold Change** | **EggNOG** | **MEGAN** | **MetaGOmics** | **MPA** | **Prophane** | **Unipept** |
| --- | --- | --- | --- | --- | --- | --- | --- |
| catalytic activity | FC (WS / NS) | 5.46 (43 / 0) | 5.16 (106 / 2) | -0.05 (30275 / 31377) | 1.67 (14.93 / 4) | 1.81 (2.5 / 0) | -0.4 (1065 / 1407.65) |
|  | Percentile (%) | 0.038 | 0.12 | 52.987 | 10.1 | 0.075 | 57.275 |
| CoA-transferase activity | FC (WS / NS) | 5.46 (43 / 0) | 1 (1 / 0) | -5.15 (15 / 568) | -1 (0 / 1) | -0.09 (0 / 0.07) | -7.84 (2 / 685.56) |
|  | Percentile (%) | 0.038 | 28.949 | 97.702 | 65.622 | 55.764 | 99.885 |
| acetate CoA-transferase activity | FC (WS / NS) | 5.46 (43 / 0) | -8.16 (0 / 285) | -6.91 (0 / 119) | - | -0.18 (0 / 0.13) | -4.37 (1 / 40.27) |
|  | Percentile (%) | 0.038 | 99.82 | 99.47 | - | 85.471 | 98.559 |
| transferase activity | FC (WS / NS) | 5.46 (43 / 0) | 5.16 (106 / 2) | -0.01 (7648 / 7717) | 3.63 (11.42 / 0) | 0.75 (0.69 / 0) | 0.01 (1789 / 1772.91) |
|  | Percentile (%) | 0.038 | 0.12 | 52.704 | 0.341 | 0.825 | 50.994 |
| transferase activity, transferring sulfur-containing groups | FC (WS / NS) | 5.46 (43 / 0) | 3.81 (13 / 0) | -2.49 (112 / 634) | - | 0.09 (0.06 / 0) | - |
|  | Percentile (%) | 0.038 | 1.321 | 81.513 | - | 20.38 | - |
| catalytic activity | FC (WS / NS) | -7.69 (0 / 206.04) | -8.59 (0 / 384) | -0.05 (30275 / 31377) | -5.29 (0 / 38) | -1.45 (0 / 1.73) | -0.4 (1065 / 1407.65) |
|  | Percentile (%) | 100 | 100 | 52.987 | 99.961 | 99.975 | 57.275 |
| glutamate dehydrogenase (NAD+) activity | FC (WS / NS) | -7.69 (0 / 206.04) | - | -3.07 (65 / 553) | - | - | -0.34 (2 / 2.81) |
|  | Percentile (%) | 100 | - | 84.765 | - | - | 56.612 |
| oxidoreductase activity | FC (WS / NS) | -7.69 (0 / 206.04) | -6.41 (0 / 84) | -0.23 (6707 / 7880) | -3.17 (0 / 8) | -0.85 (0 / 0.8) | 0.24 (2070 / 1753.24) |
|  | Percentile (%) | 100 | 99.52 | 54.118 | 95.649 | 99.375 | 42.697 |
| oxidoreductase activity, acting on the CH-NH2 group of donors | FC (WS / NS) | -7.69 (0 / 206.04) | -3 (1 / 15) | -1.43 (321 / 866) | - | -0.74 (0 / 0.67) | - |
|  | Percentile (%) | 100 | 93.393 | 71.792 | - | 99 | - |
| oxidoreductase activity, acting on the CH-NH2 group of donors, NAD or NADP as acceptor | FC (WS / NS) | -7.69 (0 / 206.04) | -1.94 (202 / 777) | -1.76 (236 / 804) | -4.32 (0 / 19) | -0.74 (0 / 0.67) | -1.81 (213 / 747.37) |
|  | Percentile (%) | 100 | 85.345 | 76.988 | 98.895 | 99 | 85.883 |

**Supplement S4 Table 3** Comparison of the top five upregulated and top five downregulated molecular function GO terms of EggNOG with the native output for molecular function GO terms from the other tools from the oral dysbiosis dataset. Fold changes are featured here (descending for EggNOG). Since eggNOG-mapper annotates on a protein level, multiple GO terms are attributed to a protein’s fold change calculation, hence the duplicate values. For other tools, if there are multiple GO terms that match the top term, then the term with the highest absolute fold change is displayed. Additionally, spectral counts are indicated for “with sucrose” and “no sucrose” (WS / NS) conditions which are used to calculate the displayed fold change (FC) = log_2_($\frac{WS + 1}{NS + 1}$). Percentiles are included to indicate the position of that particular term in that GO set containing all ontologies (0 = most upregulated; 100 = most downregulated).

A closer look at the log fold changes for molecular function GO terms from the WS and NS data (S4 Table 3) revealed that even for the same dataset there was substantial variation between functional tools. Most of the top-ranking terms from eggNOG mapper had a ranking status that was lower in other functional tools. For example, CoA-transferase activity was calculated to have a log fold change of 5.46 (0.038 percentile) while the other tools had lower log fold changes (1.00, -4.5, -1.0 and -7.84) and much lower ranks within the GO sets (percentile of 28.9, 96.1, 65.6 and 100) for MEGAN, metaGOmics, MPA and Unipept, respectively.

**Supplement S4 Table 4: MEGAN**

| **GO Term** | **Fold Change** | **MEGAN** | **EggNOG** | **MetaGOmics** | **MPA** | **Prophane** | **Unipept** |
| --- | --- | --- | --- | --- | --- | --- | --- |
| cation binding | FC (WS / NS) | 5.58 (47 / 0) | 4.75 (26 / 0) | 0.11 (13687 / 12667) | 0.91 (0.88 / 0) | 1.81 (2.5 / 0) | -0.05 (28 / 29.03) |
|  | Percentile (%) | 0.06 | 0.125 | 50.76 | 26.583 | 0.075 | 51.887 |
| metal ion binding | FC (WS / NS) | 5.58 (47 / 0) | 4.75 (26 / 0) | 0.12 (13598 / 12525) | 3.63 (11.42 / 0) | 1.81 (2.5 / 0) | 0.01 (5426 / 5382.41) |
|  | Percentile (%) | 0.06 | 0.125 | 50.442 | 0.311 | 0.075 | 51.023 |
| binding | FC (WS / NS) | 5.58 (47 / 0) | 4.75 (26 / 0) | 0.01 (31614 / 31494) | - | 1.81 (2.5 / 0) | - |
|  | Percentile (%) | 0.06 | 0.125 | 52.598 | - | 0.075 | - |
| ion binding | FC (WS / NS) | 5.58 (47 / 0) | 4.75 (26 / 0) | 0.05 (22750 / 21912) | - | 1.81 (2.5 / 0) | - |
|  | Percentile (%) | 0.06 | 0.125 | 52.033 | - | 0.075 | - |
| catalytic activity | FC (WS / NS) | 5.16 (106 / 2) | 5.46 (43 / 0) | -0.05 (30275 / 31377) | 1.67 (14.93 / 4) | 1.81 (2.5 / 0) | -0.4 (1065 / 1407.65) |
|  | Percentile (%) | 0.12 | 0.038 | 52.987 | 10.1 | 0.075 | 57.275 |
| C-acyltransferase activity | FC (WS / NS) | -8.59 (0 / 384) | -6.45 (0 / 86.16) | -1.68 (230 / 738) | - | -1.09 (0 / 1.13) | - |
|  | Percentile (%) | 100 | 99.913 | 75.716 | - | 99.825 | - |
| acetyl-CoA C-acyltransferase activity | FC (WS / NS) | -8.59 (0 / 384) | -6.45 (0 / 86.16) | -8.37 (0 / 330) | -5 (0 / 31) | -1.09 (0 / 1.13) | -7.92 (0 / 240.7) |
|  | Percentile (%) | 100 | 99.913 | 99.894 | 99.819 | 99.825 | 99.914 |
| acetyltransferase activity | FC (WS / NS) | -8.59 (0 / 384) | -6.45 (0 / 86.16) | -0.85 (557 / 1004) | -4.91 (0 / 29) | -1.09 (0 / 1.13) | 0.22 (217 / 186.38) |
|  | Percentile (%) | 100 | 99.913 | 64.723 | 99.715 | 99.825 | 42.927 |
| C-acetyltransferase activity | FC (WS / NS) | -8.59 (0 / 384) | -6.45 (0 / 86.16) | -1.68 (230 / 738) | - | -1.09 (0 / 1.13) | - |
|  | Percentile (%) | 100 | 99.913 | 75.751 | - | 99.825 | - |
| acetyl-CoA C-acetyltransferase activity | FC (WS / NS) | -8.59 (0 / 384) | -6.45 (0 / 86.16) | -8.37 (0 / 330) | -5.46 (0 / 43) | -1.09 (0 / 1.13) | -8.54 (0 / 369.94) |
|  | Percentile (%) | 100 | 99.913 | 99.859 | 99.987 | 99.825 | 100 |

**Supplement S4 Table 4** Comparison of the top five upregulated and top five downregulated molecular function GO terms of MEGAN with the native output for molecular function GO terms from the other tools from the oral dysbiosis dataset. Fold changes are featured here (descending for MEGAN). Since MEGAN annotates on a protein level, multiple GO terms are attributed to a protein’s fold change calculation, hence the duplicate values. Since MEGAN annotates via protein, multiple GO terms are attributed to a protein’s fold change calculation, hence the duplicate values. For other tools, if there are multiple GO terms that match the top term, then the term with the highest absolute fold change is displayed. Additionally, spectral counts are indicated for “with sucrose” and “no sucrose” (WS / NS) conditions which are used to calculate the displayed fold change (FC) = log_2_($\frac{WS + 1}{NS + 1}$). Percentiles are included to indicate the position of that particular term in that GO set containing all ontologies (0 = most upregulated; 100 = most downregulated).

MEGAN’s top terms originate from the same COG and seem to match well with EggNOG-mapper. The fold changes are similar, but the spectral count ratios are proportionally higher in MEGAN. Additionally, EggNOG-Mapper’s matched terms also originate from the same protein. These term groups are also apparently ancestor/descendant terms of one another and are both captured by both tools. For the other tools, however, fold changes do not match as well. Exceptions include the downregulated terms of MetaGOmics and MPA and the upregulated term ‘metal ion binding’ in MPA.

**Supplement S4 Table 5: MetaGOmics**

| **GO Term** | **Fold Change** | **MetaGOmics** | **EggNOG** | **MEGAN** | **MPA** | **Prophane** | **Unipept** |
| --- | --- | --- | --- | --- | --- | --- | --- |
| dextransucrase activity | FC (WS / NS) | 6.71 (104 / 0) | - | - | 3.01 (7.03 / 0) | - | 6.67 (101 / 0) |
|  | Percentile (%) | 0.035 | - | - | 1.157 | - | 0.086 |
| pyruvate oxidase activity | FC (WS / NS) | 6.13 (69 / 0) | - | - | - | -0.09 (0 / 0.07) | 6.38 (82 / 0) |
|  | Percentile (%) | 0.071 | - | - | - | 57.689 | 0.115 |
| oxidoreductase activity, acting on the aldehyde or oxo group of donors, oxygen as acceptor | FC (WS / NS) | 6.13 (69 / 0) | - | - | - | -0.09 (0 / 0.07) | - |
|  | Percentile (%) | 0.106 | - | - | - | 57.689 | - |
| glyceraldehyde-3-phosphate dehydrogenase (NADP+) (non-phosphorylating) activity | FC (WS / NS) | 6.02 (64 / 0) | - | - | - | - | 6.04 (65 / 0) |
|  | Percentile (%) | 0.141 | - | - | - | - | 0.144 |
| glyceraldehyde-3-phosphate dehydrogenase (NADP+) (phosphorylating) activity | FC (WS / NS) | 6.02 (64 / 0) | - | - | - | - | - |
|  | Percentile (%) | 0.177 | - | - | - | - | - |
| beta-lysine 5,6-aminomutase activity | FC (WS / NS) | -7.54 (0 / 185) | - | - | - | -1 (0 / 1) | -7.27 (0 / 153.6) |
|  | Percentile (%) | 99.753 | - | - | - | 99.575 | 99.827 |
| lysine 2,3-aminomutase activity | FC (WS / NS) | -7.74 (0 / 213) | - | -7.29 (1 / 311) | -5 (0 / 31) | -0.62 (0 / 0.53) | -7.13 (1 / 280.03) |
|  | Percentile (%) | 99.788 | - | 99.7 | 99.81 | 98.675 | 99.798 |
| glutaconate CoA-transferase activity | FC (WS / NS) | -8.13 (0 / 279) | - | - | -4 (0 / 15) | -0.55 (0 / 0.47) | -8.41 (0 / 339.03) |
|  | Percentile (%) | 99.823 | - | - | 98.412 | 98.25 | 99.971 |
| acetyl-CoA C-acetyltransferase activity | FC (WS / NS) | -8.37 (0 / 330) | -6.45 (0 / 86.16) | -8.59 (0 / 384) | -5.46 (0 / 43) | -1.09 (0 / 1.13) | -8.54 (0 / 369.94) |
|  | Percentile (%) | 99.859 | 99.913 | 100 | 99.987 | 99.825 | 100 |
| acetyl-CoA C-acyltransferase activity | FC (WS / NS) | -8.37 (0 / 330) | -6.45 (0 / 86.16) | -8.59 (0 / 384) | -5 (0 / 31) | -1.09 (0 / 1.13) | -7.92 (0 / 240.7) |
|  | Percentile (%) | 99.894 | 99.913 | 100 | 99.819 | 99.825 | 99.914 |

**Supplement S4 Table 5** Comparison of the top five upregulated and top five downregulated molecular function GO terms of metaGOmics with native output for the molecular function GO terms from the other tools from the oral dysbiosis dataset. Fold changes are featured here (descending for metaGOmics). Since eggNOG-mapper annotates on a protein level, multiple GO terms are attributed to a protein’s fold change calculation, hence the duplicate values. For other tools, if there are multiple GO terms that match the top term, then the term with the highest absolute fold change is displayed. Additionally, spectral counts are indicated for “with sucrose” and “no sucrose” (WS / NS) conditions which are used to calculate the displayed fold change (FC) = log_2_($\frac{WS + 1}{NS + 1}$). Percentiles are included to indicate the position of that particular term in that GO set containing all ontologies (0 = most upregulated; 100 = most downregulated).

For MetaGOmics, only one of its top upregulated terms showed similarity to other tools (S4 Table 5). Generally, these terms were not found in most of the other tools. One exception is dextransucrase activity which has a log fold change of 6.71 (0.035 percentile) in MetaGOmics and is also found in MPA (fold-change 3.01 and percentile of 1.157). For down-regulated GO terms the overlap was much better, though not complete. For example, for acetyl-CoA C-acyltransferase the percentile values for metaGOmics, eggNOG-mapper, MEGAN and MetaProteomeAnalyzer were more than 99.8.

**Supplement S4 Table 6: MetaProteomeAnalyzer**

| **GO Term** | **Fold Change** | **MPA** | **EggNOG** | **MEGAN** | **MetaGOmics** | **Prophane** | **Unipept** |
| --- | --- | --- | --- | --- | --- | --- | --- |
| ATP binding | FC (WS / NS) | 4.35 (19.33 / 0) | 4.46 (21 / 0) | 4.09 (16 / 0) | 0.27 (9736 / 8100) | 1.06 (1.5 / 0.2) | 0.19 (11853 / 10405.17) |
|  | Percentile (%) | 0.086 | 0.211 | 1.021 | 46.235 | 0.325 | 43.359 |
| unfolded protein binding | FC (WS / NS) | 4.35 (19.33 / 0) | 4.46 (21 / 0) | - | 0.46 (1380 / 1003) | 0.75 (0.69 / 0) | 0.63 (1769 / 1139.79) |
|  | Percentile (%) | 0.086 | 0.211 | - | 39.661 | 0.875 | 34.947 |
| lyase activity | FC (WS / NS) | 2.84 (6.15 / 0) | 3.81 (13 / 0) | 5.16 (106 / 2) | -0.5 (3220 / 4543) | 0.7 (1.06 / 0.27) | -0.31 (936 / 1160.4) |
|  | Percentile (%) | 1.493 | 0.738 | 0.12 | 57.37 | 1.025 | 55.287 |
| IMP dehydrogenase activity | FC (WS / NS) | 2.84 (6.15 / 0) | 3.7 (12 / 0) | -0.65 (13 / 21) | - | 0.49 (0.5 / 0.07) | -0.15 (145 / 161.09) |
|  | Percentile (%) | 1.506 | 0.841 | 60.901 | - | 2.251 | 53.097 |
| fumarate hydratase activity | FC (WS / NS) | 2.84 (6.15 / 0) | 3.32 (9 / 0) | 1.96 (237 / 60) | 0.53 (148 / 102) | 0.52 (0.44 / 0) | 1.2 (414 / 179.82) |
|  | Percentile (%) | 1.619 | 1.871 | 10.751 | 36.479 | 2.101 | 17.257 |
| metalloaminopeptidase activity | FC (WS / NS) | -3.32 (0 / 9) | -0.95 (0 / 0.94) | - | -2.03 (20 / 85) | - | -2.18 (19 / 89.91) |
|  | Percentile (%) | 96.418 | 72.76 | - | 79.215 | - | 91.011 |
| lyase activity | FC (WS / NS) | -4.58 (0 / 23) | -5.79 (0 / 54.32) | -7.16 (2 / 427) | -0.5 (3220 / 4543) | -1.25 (0.06 / 1.53) | -0.31 (936 / 1160.4) |
|  | Percentile (%) | 99.413 | 99.74 | 99.64 | 57.37 | 99.9 | 55.287 |
| electron transfer activity | FC (WS / NS) | -5.43 (0 / 42) | -4.43 (0 / 20.6) | -6.41 (0 / 84) | -1.16 (678 / 1516) | -1.45 (0 / 1.73) | -1.03 (774 / 1584.66) |
|  | Percentile (%) | 99.983 | 98.514 | 99.52 | 68.363 | 99.95 | 75.799 |
| ligase activity | FC (WS / NS) | -4.52 (0 / 22) | -5.71 (0 / 51.51) | -5.32 (0 / 39) | 0.12 (3619 / 3335) | -0.79 (0 / 0.73) | -0.42 (387 / 519.79) |
|  | Percentile (%) | 99.219 | 99.707 | 98.859 | 50.513 | 99.3 | 57.505 |
| tryptophanase activity | FC (WS / NS) | -4.58 (0 / 23) | - | -7.16 (2 / 427) | -7.41 (0 / 169) | -1.05 (0 / 1.07) | -6.6 (1 / 192.93) |
|  | Percentile (%) | 99.37 | - | 99.64 | 99.717 | 99.65 | 99.77 |

**Supplement S4 Table 6:** Comparison of the top five upregulated and top five downregulated molecular function GO terms of MPA with the native output for molecular function GO terms from the other tools from the oral dysbiosis dataset. Fold changes are featured here (descending for MPA). Since MPA annotates on a protein level, multiple GO terms are attributed to a protein’s fold change calculation, hence the duplicate values. For other tools, if there are multiple GO terms that match the top term, then the term with the highest absolute fold change is displayed. Additionally, spectral counts are indicated for “with sucrose” and “no sucrose” (WS / NS) conditions which are used to calculate the displayed fold change (FC) = log_2_($\frac{WS + 1}{NS + 1}$). Percentiles are included to indicate the position of that particular term in that GO set containing all ontologies (0 = most upregulated; 100 = most downregulated).

For MetaProteomeAnalyzer, most of the top upregulated and downregulated GO terms overlapped well with other tools. Quantitatively, MPA had similar fold changes in EggNOG-mapper and MEGAN, but not the other three tools. For example, MPA, EggNOG-mapper and MEGAN attributed zero spectral counts for the NS condition for ATP binding (top upregulated term) while metaGOmics and Unipept had upwards of 8,000 spectral counts.

**Supplement S4 Table 7: ProPHAnE**

| **GO Term** | **Fold Change** | **Prophane** | **EggNOG** | **MEGAN** | **MetaGOmics** | **MPA** | **Unipept** |
| --- | --- | --- | --- | --- | --- | --- | --- |
| structural constituent of ribosome | FC (WS / NS) | 1.81 (3.69 / 0.33) | 5.43 (42 / 0) | 2 (3 / 0) | -0.18 (5008 / 5685) | 3.63 (11.42 / 0) | -0.1 (5890 / 6308.66) |
|  | Percentile (%) | 0.05 | 0.043 | 9.61 | 53.517 | 0.319 | 52.348 |
| structural molecule activity | FC (WS / NS) | 1.81 (3.69 / 0.33) | 5.43 (42 / 0) | 2 (3 / 0) | -0.24 (5131 / 6058) | 2.65 (5.27 / 0) | -1.18 (179 / 407.4) |
|  | Percentile (%) | 0.05 | 0.043 | 9.61 | 54.224 | 2.18 | 76.721 |
| ferric iron binding | FC (WS / NS) | 1.81 (2.5 / 0) | -0.52 (1 / 1.87) | 1.52 (411 / 143) | 0.44 (374 / 276) | 5.65 (49.19 / 0) | 0.43 (476 / 352.15) |
|  | Percentile (%) | 0.075 | 57.088 | 18.018 | 40.933 | 0.004 | 39.672 |
| nucleic acid binding | FC (WS / NS) | 1.81 (2.5 / 0) | 4.64 (24 / 0) | 4 (15 / 0) | -0.16 (9968 / 11155) | 3.41 (9.66 / 0) | -0.1 (1032 / 1106.08) |
|  | Percentile (%) | 0.075 | 0.157 | 1.201 | 53.411 | 0.423 | 52.377 |
| DNA binding | FC (WS / NS) | 1.81 (2.5 / 0) | 3.17 (8 / 0) | 3.92 (150 / 9) | 0.1 (2311 / 2155) | 5.65 (49.19 / 0) | 0.02 (3961 / 3912.94) |
|  | Percentile (%) | 0.075 | 2.023 | 1.261 | 51.043 | 0.004 | 50.85 |
| aspartic-type endopeptidase activity | FC (WS / NS) | -1.45 (0 / 1.73) | - | 2.81 (6 / 0) | - | - | 0.73 (24 / 14.05) |
|  | Percentile (%) | 99.975 | - | 4.745 | - | - | 33.564 |
| peptidase activity | FC (WS / NS) | -1.45 (0 / 1.73) | -5.16 (0 / 34.65) | -5.83 (0 / 56) | 0.32 (1575 / 1258) | -3 (0 / 7) | 1.65 (968 / 308.13) |
|  | Percentile (%) | 99.975 | 99.366 | 99.279 | 44.68 | 94.065 | 10.573 |
| hydrolase activity | FC (WS / NS) | -1.45 (0 / 1.73) | -5.58 (0 / 46.83) | -5.83 (0 / 56) | 0.1 (9209 / 8591) | -3.58 (0 / 11) | 0.07 (1060 / 1010.55) |
|  | Percentile (%) | 99.975 | 99.61 | 99.279 | 51.078 | 97.583 | 45.837 |
| aspartic-type peptidase activity | FC (WS / NS) | -1.45 (0 / 1.73) | - | 2.81 (6 / 0) | - | - | - |
|  | Percentile (%) | 99.975 | - | 4.745 | - | - | - |
| peptidase activity, acting on L-amino acid peptides | FC (WS / NS) | -1.45 (0 / 1.73) | -5.16 (0 / 34.65) | -5.83 (0 / 56) | 0.32 (1555 / 1245) | -1 (0 / 1) | -2.92 (0 / 6.56) |
|  | Percentile (%) | 99.975 | 99.366 | 99.279 | 44.821 | 68.514 | 95.736 |

**Supplement S4 Table 7** Comparison of the top five upregulated and top five downregulated molecular function GO terms of ProPHAnE with native output for the molecular function GO terms from the other tools from the oral dysbiosis dataset. Fold changes are featured here (descending for ProPHAnE). Since ProPHAnE annotates on a protein level, multiple GO terms are attributed to a protein’s fold change calculation, hence the duplicate values. For other tools, if there are multiple GO terms that match the top term, then the term with the highest absolute fold change is displayed. Additionally, spectral counts are indicated for “with sucrose” and “no sucrose” (WS / NS) conditions which are used to calculate the displayed fold change (FC) = log_2_($\frac{WS + 1}{NS + 1}$). Percentiles are included to indicate the position of that particular term in that GO set containing all ontologies (0 = most upregulated; 100 = most downregulated).

A closer look at ProPHAnE’s top terms revealed that there is limited quantitative information from ProPHAnE due to reduced quantitative information due to a different output (not spectral counts). However, regardless of this difference, there is an agreement percentile-wise with all tools except for the GO-level tools (MetaGOmics and Unipept).

For all the tools, all the raw, expanded and filtered outputs have been provided in the updated Zenodo link (https://zenodo.org/record/3816624)

**Supplement S5**

**
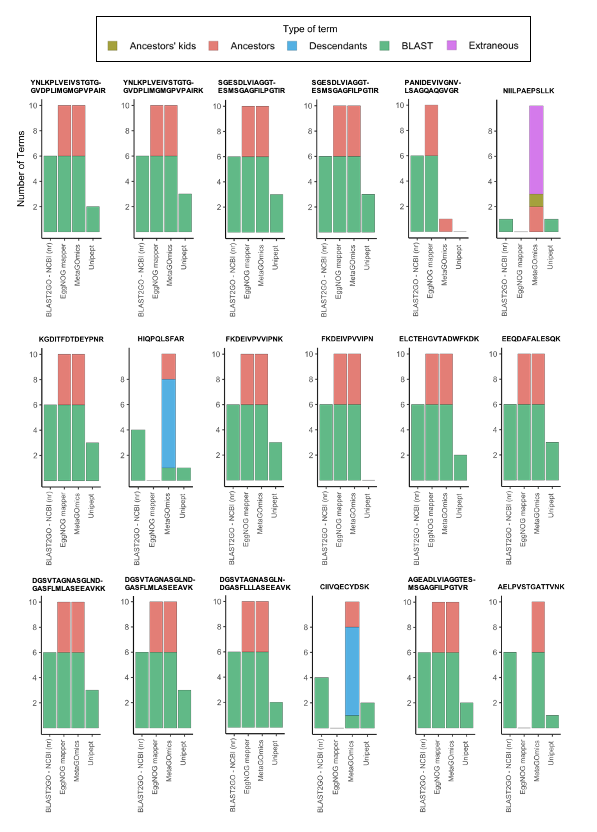
**

Distribution of molecular function GO term ‘Acetyl-CoA C-acetyltransferase activity’ for eggNOG mapper, metaGOmics and Unipept for 18 randomly selected peptides from the oral dysbiosis dataset. Molecular function GO terms found in common with BLAST NCBI (nr) were set as an anchor point at the bottom (green). The rest of the GO terms were hierarchically categorized relative to these BLAST NCBI(nr)-intersected GO terms e.g., descendants (blue) and ancestors (orange). Ancestors’ kids are any terms that are the direct descendants of any ancestors of any BLAST/Unipept-intersected GO term in each peptide.

**Supplement S6
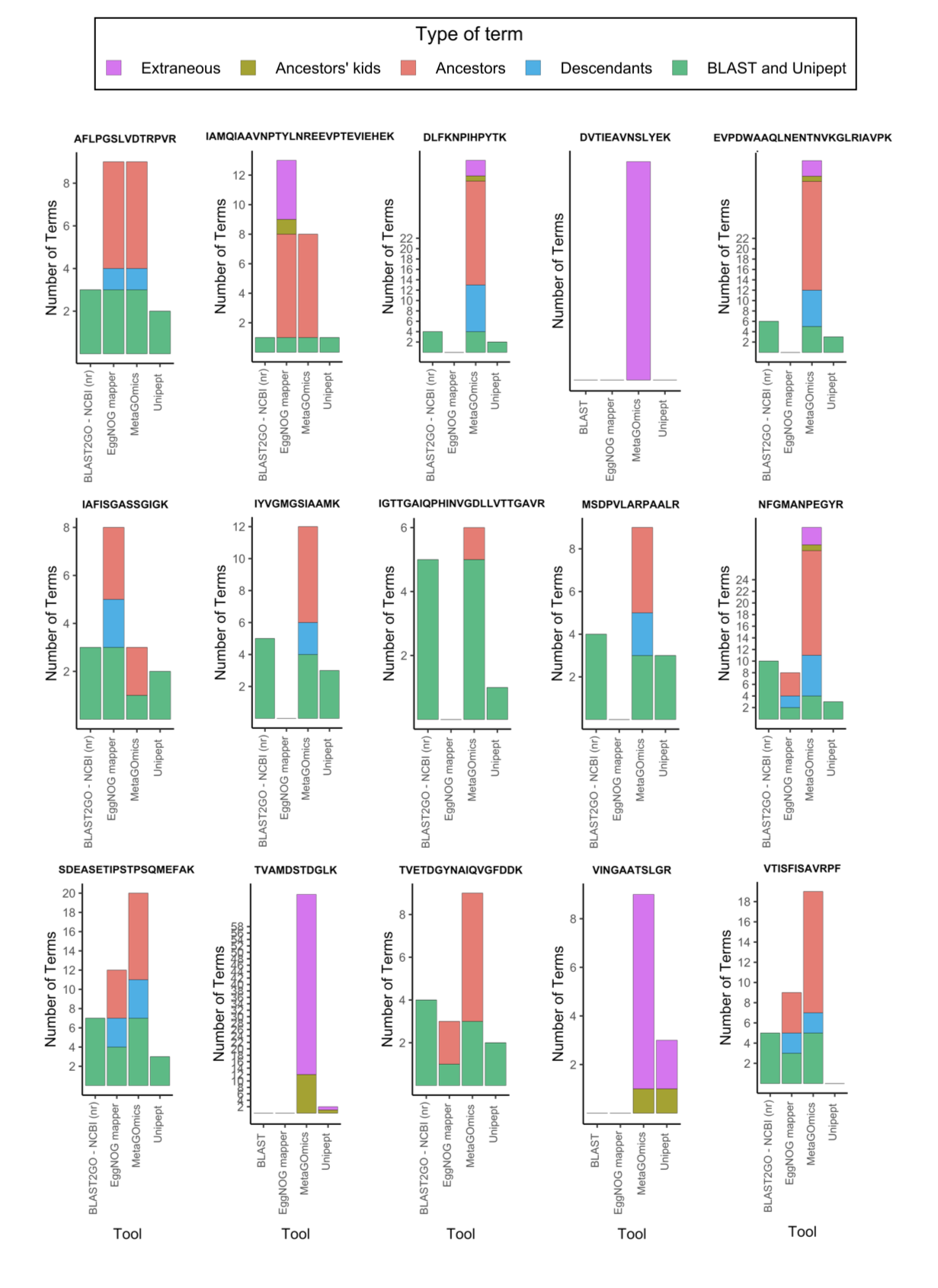
**

Distribution of molecular function GO terms of eggNOG mapper, metaGOmics, and Unipept for 15 randomly selected peptides from the oral dysbiosis dataset. Molecular function GO terms found in common with BLAST NCBI (nr) were set as an anchor point at the bottom. The rest of the GO terms were hierarchically categorized relative to these BLAST/Unipept-intersected GO terms e.g., descendants and ancestors. Ancestors’ kids are any terms that are the direct descendants of any ancestors of any BLAST/Unipept-intersected GO term in each peptide. Extraneous terms are any terms that do not fit in any of the aforementioned categories.

**Supplement S7**


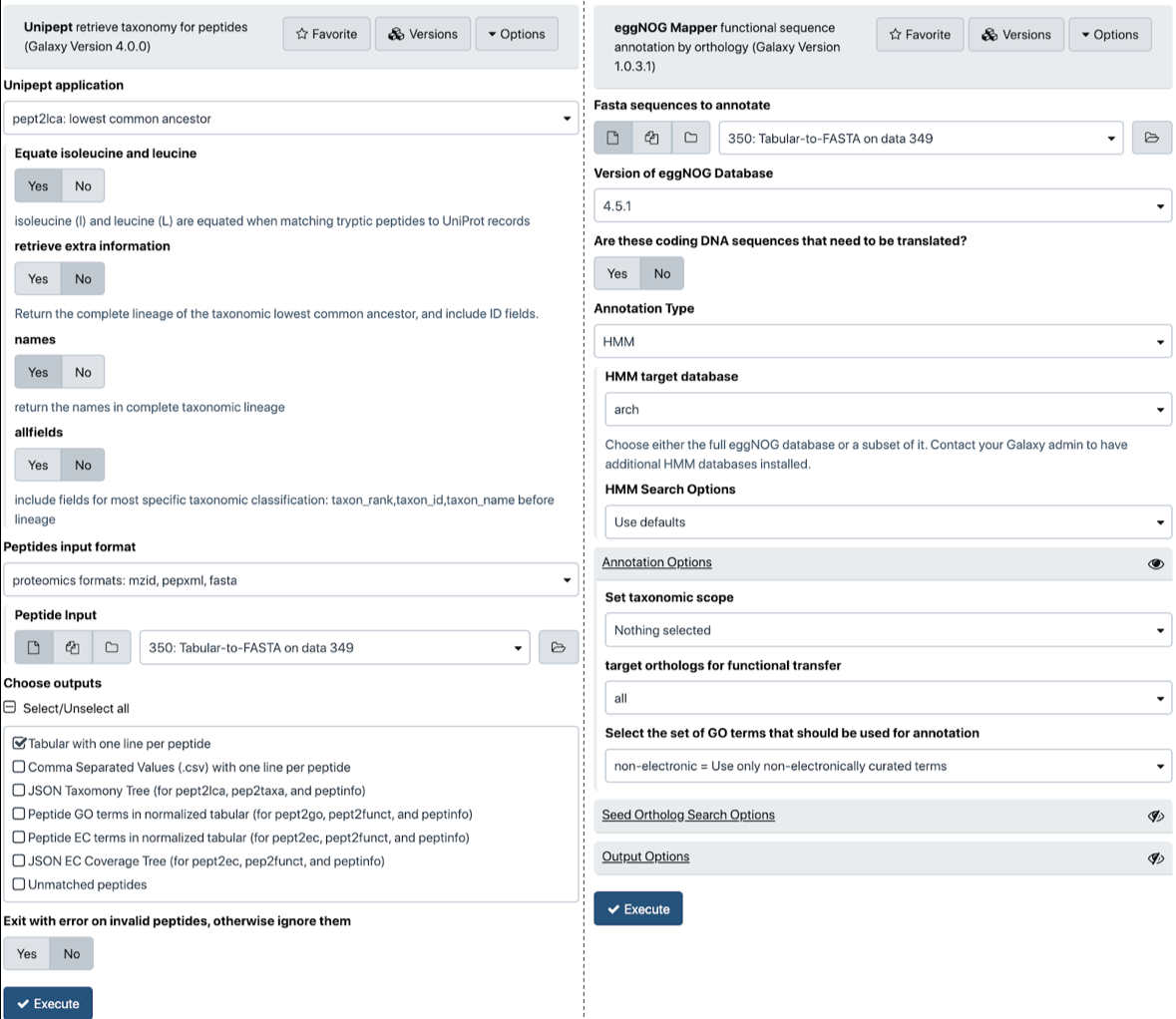


**A) Galaxy interface of Unipept 4.0:** Unipept 4.0 is wrapped within Galaxy and available via Galaxy toolshed (<https://toolshed.g2.bx.psu.edu/view/galaxyp/unipept>), GitHub (https://github.com/galaxyproteomics/tools-galaxyp/tree/master/tools/unipept) and via Galaxy public instances (usegalaxy.eu and z.umn.edu/metaproteomicsgateway).

**B) Galaxy interface of eggNOG-mapper:** eggNOG-mapper is wrapped within Galaxy and available via Galaxy toolshed (<https://toolshed.g2.bx.psu.edu/view/galaxyp/eggnog_mapper/>), GitHub (https://github.com/galaxyproteomics/tools-galaxyp/tree/master/tools/eggnog_mapper) and Galaxy public instances (usegalaxy.eu and z.umn.edu/metaproteomicsgateway).

**Galaxy implementation:** While assessing the performance of the functional tools presented in this study, we also worked with software developers to explore the possibility of making some of these tools available to metaproteomics researchers within scalable computing environments. To this end, we have implemented software tools and workflows within the Galaxy platform to enable multi-omics analysis. EggNOG-mapper uses orthology assignment, wherein the best matching peptide sequences associated with a protein in the eggNOG database is used to retrieve orthology assignments. In this step, paralogs and matches without sufficient homology are excluded. Subsequently, functions for the retrieved orthologs are transferred to the corresponding query proteins. For eggNOG-mapper implementation within Galaxy, the eggNOG database has to be made accessible along with the interface. The Galaxy data manager application makes it easier for a Galaxy administrator to install reference data. For its usage in Galaxy, users can use DIAMOND against the eggNOG database to generate seed orthologs. The eggNOG-mapper software tool generates outputs such as HMM-hits annotation (if HMM is used as a method) and DIAMOND seed orthologs and annotation (if DIAMOND used as a method).The Galaxy version of eggNOG-mapper is available via Galaxy toolshed (https://toolshed.g2.bx.psu.edu/view/galaxyp/eggnog_mapper/), GitHub (https://github.com/galaxyproteomics/tools-galaxyp/tree/master/tools/eggnog_mapper) and Galaxy public instances (usegalaxy.eu and z.umn.edu/metaproteomicsgateway).

The Unipept software can take in multiple peptide sequences and match them against UniProt database to retrieve taxonomy and functional information. The Unipept Galaxy tool retrieves taxonomy classification and protein-related functional information for peptides using the Unipept API (https://unipept.ugent.be/apidocs). The Unipept tool can read query peptides from a fasta, mzxml, pepxml, or tabular file. The tool has an associated python script that performs the API interaction. (https://github.com/galaxyproteomics/tools-galaxyp/blob/master/tools/unipept/unipept.py) It splits all query peptides into tryptic peptides, queries the Unipept API with the tryptic peptides, then matches the assignments back to the query peptides, generating a tabular format output. Additionally, the output can be a JSON file that can be viewed by the galaxy Unipept tree view visualization(https://github.com/galaxyproteomics/tools-galaxyp/tree/master/visualizations/unipept) which is an adaptation of the Unipept tree view visualization(https://github.com/unipept/unipept-visualizations). Within Unipept, a user can perform several kinds of peptide-level analysis including a) access UniProt accession numbers for peptides (pept2prot); b) access taxa information from UniProt entries (pept2taxa and pept2lca); c) access protein EC numbers (pept2ec) and d) access GO term for the peptides (pept2GO). Additionally, Unipept also generates JSON tree outputs that can be used to interactively visualize taxonomic information and EC proteins identified in the sample. Galaxy version of Unipept 4.0 is available via Galaxy toolshed (https://toolshed.g2.bx.psu.edu/view/galaxyp/unipept), GitHub (https://github.com/galaxyproteomics/tools-galaxyp/tree/master/tools/unipept) and via Galaxy public instances (usegalaxy.eu and z.umn.edu/metaproteomicsgateway).

**Supplement S8**

The runtimes of Galaxy-implemented tools that ran on the same server (usegalaxy.eu). Galaxy (v19.09) offers other job metric information as well.

|  | **EggNOG-mapper** | **Unipept** |
| --- | --- | --- |
| **Memory Allocated (MB)** | 4096 | 4096 |
| **CPU Time** | 3.1 hours and 5.79 minutes | 36.67 seconds |
| **Max memory usage (MEM)** | 7.8 GB | 1.1 GB |
| **Cores Allocated** | 1 | 1 |
| **Runtime** | 20.0 hours and 36.0 minutes | 240 seconds |
