# Supplementary material for: Survey of metaproteomics software tools for functional microbiome analysis
Source: PLoS One. 2020 Nov 10;15(11):e0241503. doi: 10.1371/journal.pone.0241503 (PMC7654790; doi:10.1371/journal.pone.0241503)
Supplement: Attachment — Submitted filename: Response to Reviewers PONE-D-20-04130.docx [file pone.0241503.s002.docx]

PONE-D-20-04130

Survey of metaproteomics software tools for functional microbiome analysis.

PLOS ONE

Dear Dr Jagtap,

Thank you for submitting your manuscript to PLOS ONE. After careful consideration, we feel that it has merit but does not fully meet PLOS ONE’s publication criteria as it currently stands. Therefore, we invite you to submit a revised version of the manuscript that addresses the points raised during the review process.

The overall assessment of this work is positive but reviewers have raised several issues that need to be addressed. Regarding the comparison and presentation of the results, the choice of Galaxy should be better justified. Including the run time would also enhance the results. In-depth arguments are also required in particular regarding the discussion raised by reviewer1 on the use of GO terms. Careful accounting of the reviewers' comments can definitely make this paper a flagship for users of metaproteomics analysis pipelines.

**We thank the editor for the comments. Based on the reviewer’s inputs, we have made changes to the manuscript. We believe the changes based on the reviewer feedback has substantially improved the quality of the manuscript.**

We would appreciate receiving your revised manuscript by April 30, 2020. When you are ready to submit your revision, log on to <https://www.editorialmanager.com/pone/> and select the 'Submissions Needing Revision' folder to locate your manuscript file.

If you would like to make changes to your financial disclosure, please include your updated statement in your cover letter.

To enhance the reproducibility of your results, we recommend that if applicable you deposit your laboratory protocols in [protocols.io](http://protocols.io/), where a protocol can be assigned its own identifier (DOI) such that it can be cited independently in the future. For instructions see: <http://journals.plos.org/plosone/s/submission-guidelines#loc-laboratory-protocols>

Please include the following items when submitting your revised manuscript:

- A rebuttal letter that responds to each point raised by the academic editor and reviewer(s). This letter should be uploaded as separate file and labeled 'Response to Reviewers'.
- A marked-up copy of your manuscript that highlights changes made to the original version. This file should be uploaded as separate file and labeled 'Revised Manuscript with Track Changes'.
- An unmarked version of your revised paper without tracked changes. This file should be uploaded as separate file and labeled 'Manuscript'.

Please note while forming your response, if your article is accepted, you may have the opportunity to make the peer review history publicly available. The record will include editor decision letters (with reviews) and your responses to reviewer comments. If eligible, we will contact you to opt in or out.

We look forward to receiving your revised manuscript.

Kind regards,

Frederique Lisacek

Academic Editor

PLOS ONE

Journal Requirements:

When submitting your revision, we need you to address these additional requirements:

1. Please ensure that your manuscript meets PLOS ONE's style requirements, including those for file naming. The PLOS ONE style templates can be found at <http://www.plosone.org/attachments/PLOSOne_formatting_sample_main_body.pdf> and <http://www.plosone.org/attachments/PLOSOne_formatting_sample_title_authors_affiliations.pdf>

2.Please ensure that you refer to Figure 4 in your text as, if accepted, production will need this reference to link the reader to the figure.

**Based on the inputs from Reviewer 2, we have deleted Figure 4 from the manuscript and moved it to the supplement section (Supplement S7).**

3. We note that you have included the phrase “data not shown” in your manuscript. Unfortunately, this does not meet our data sharing requirements. PLOS does not permit references to inaccessible data. We require that authors provide all relevant data within the paper, Supporting Information files, or in an acceptable, public repository. Please add a citation to support this phrase or upload the data that corresponds with these findings to a stable repository (such as Figshare or Dryad) and provide and URLs, DOIs, or accession numbers that may be used to access these data. Or, if the data are not a core part of the research being presented in your study, we ask that you remove the phrase that refers to these data.

**We have deleted the following sentence from the current version of the manuscript. *“We found that this was also the case for single organism peptides wherein functional annotation depends on matching with a single organism GO term database (data not shown).”***

4. Please include captions for your Supporting Information files at the end of your manuscript, and update any in-text citations to match accordingly. Please see our Supporting Information guidelines for more information: <http://journals.plos.org/plosone/s/supporting-information>.

[Note: HTML markup is below. Please do not edit.]

Reviewers' comments:

Reviewer's Responses to Questions

**Comments to the Author**

1. Is the manuscript technically sound, and do the data support the conclusions?

The manuscript must describe a technically sound piece of scientific research with data that supports the conclusions. Experiments must have been conducted rigorously, with appropriate controls, replication, and sample sizes. The conclusions must be drawn appropriately based on the data presented.

Reviewer #1: Partly

Reviewer #2: Yes

2. Has the statistical analysis been performed appropriately and rigorously?

Reviewer #1: Yes

Reviewer #2: No

3. Have the authors made all data underlying the findings in their manuscript fully available?

The [PLOS Data policy](http://www.plosone.org/static/policies.action#sharing) requires authors to make all data underlying the findings described in their manuscript fully available without restriction, with rare exception (please refer to the Data Availability Statement in the manuscript PDF file). The data should be provided as part of the manuscript or its supporting information, or deposited to a public repository. For example, in addition to summary statistics, the data points behind means, medians and variance measures should be available. If there are restrictions on publicly sharing data—e.g. participant privacy or use of data from a third party—those must be specified.

Reviewer #1: No

Reviewer #2: No

4. Is the manuscript presented in an intelligible fashion and written in standard English?

PLOS ONE does not copyedit accepted manuscripts, so the language in submitted articles must be clear, correct, and unambiguous. Any typographical or grammatical errors should be corrected at revision, so please note any specific errors here.

Reviewer #1: Yes

Reviewer #2: Yes

5. Review Comments to the Author

Please use the space provided to explain your answers to the questions above. You may also include additional comments for the author, including concerns about dual publication, research ethics, or publication ethics. (Please upload your review as an attachment if it exceeds 20,000 characters)

Reviewer #1: The manuscript aims to benchmark six available metaproteome annotation tools, eggNOG-mapper, MEGAN6, MetaGOmics, MetaProteomeAnalyzer (MPA), ProPHAnE, and Unipept.

To benchmark the tools, an “oral dysbiosis” dataset was selected which includes the samples of plaque bacteria that were grown with and without sucrose. For both of these samples, the predicted peptides were normalised and mapped to functional groups/terms using the benchmarked tools. Subsequently the differential expression of the terms were calculated for each of the tool. The core of the benchmark is the comparison of these differentially expressed terms.

The manuscript also extensively compares inputs and outputs of the benchmarked tools. As they vary widely in that respect, the presented methodology generates specific inputs for each of the tools and aims to synthesise comparable output datasets.

The authors find that although the differentially expressed annotated terms are all significantly correlated between the tools, they exhibit a large degree of variability. At the end two of the tools (one which exhibits high specificity and sensitivity, and one that mapped the largest amount of terms) were then incorporated into the Galaxy platform.

Growing interest in and availability of metaproteomics data will result in significant need for annotation tools and established annotation pipelines. The presented manuscript could be of significant interest to metaproteomics community and have immediate impact on metaproteomics analysis methods. Therefore it is important to assure that the analysis presented in the manuscript is correct and interpretable.

The core aspect of the presented methodology is assigning the differential expression to each GO term and making the sets comparable between the tools. Unfortunately this is also the weakest part of the manuscript. For the methods to be comparable, each GO term, for each peptide assigned by the method, should be projected to the root of its ontology and the differential expression should be calculated at each of the GO terms after that projection. It is not clear if this is done here. As such, without this projection, the methods that assign more granular terms will have diminished chance of picking up the signal, as the differential expression signal would be divided onto the term’s children whereas less granular method could incorporate the signal from all the term’s children. GO prediction benchmarking should reward the methods that assign more granular terms, as the GO terms could always be projected. Instead it seems that described method of the comparison effectively punishes for it. This is such a critical weakness, that lets me to believe I did not understand the methodology correctly. However the only part of the manuscript that describes the implementation of GO ontology traversal is found in the analysis of the 20 random peptides which is not related to the core of the comparison.

**We thank the reviewer for the suggestion. In order to address this concern, we used the expand and filter options within the metaQuantome tool. In particular this method helped us to extend the number of GO terms for tools that reported specific and limited number of terms (Figures 2A and table 1 in the main text) and incorporate a subsequent filtering step (S4 tables in the supplementary section) that ensured that we compared all the terms at the same basal level. We have modified Table 2 to report the Top 5 up-regulated GO terms and top 5 down-regulated GO terms. The extended version and extended and filtered version of Table 2, have been added as Supplement S4 Table 1 and Table 2 in the supplementary section.**

**As a result of the expansion and filtering, the fold-change for the WS/NS was reduced and apart from the two up-regulated GO-terms and three down-regulated GO terms, rankings (denoted by percentile) for Unipept have changed. As a result of expansion and filtering, we also saw a decreased overlap especially for MetaProteomeAnalyzer and EggNOG mapper. This result demonstrates that in spite of using the expand and filter option, we do not see a good overlap amongst the differentially expressed GO terms from tools based on the fold-change and percentile values. We have decided to retain the table with unfiltered values in the main text while providing readers an option to investigate the effect of expansion and filtering on the fold-change and ranking of the differentially GO terms (Supplement S4 Table 1 and Table 2). We have also used expansion of GO terms in other figures such as Table 1, Figure 2A, Supplement S1 Tables 1-3; Supplement S2 Figures 2-5; Supplement S4 Tables 1-2. We have used filtered terms only for S2 and S4. We also provide the entire list of GO terms for all the six software tools in unfiltered, expanded and filtered form as Excel sheets in the updated Zenodo link (https://zenodo.org/record/3816624).**

To partially mitigate the above point GO terms were translated into higher level GO slim subset, however this subset consists of only 147 terms, which may be too broad to generate the interpretable differential expression signal. I say potentially, as authors did not include in the manuscript any results from GO slim comparison except calculating Jaccard index between the tools’ outputs, which is not particularly informative.

**We agree with the reviewer’s comments and have not used GO Slim output in Figure 2 (Overlap of unique molecular function GO terms). We have instead added the expand feature to compare the GO terms across the tools and see an improvement in the overlap due to extension of GO terms (Supplement S4).**

All differentially expressed list of terms (projected to root) from all the tested methods, for all three GO ontologies should be included as a part of supplementary data. This will allow readers a way to judge the validity of the method by themselves. The overlap comparisons and “Top ten” terms for each method not sufficient for a meaningful interpretation.

**We have provided the list of expanded and filtered terms for all three GO ontologies along with fold-changes and associated percentile scores (see Zenodo link: https://zenodo.org/record/3816624). We provide the readers an opportunity to investigate the top five up-regulated and down-regulated raw outputs for molecular function GO ontologies for Unipept in comparison with other software tools (Table 2), along with an expanded molecular function GO ontologies (Supplement S4 Table 1) and expanded and filtered molecular function GO ontologies (Supplement S4 Table 2).**

Another point that should be addressed is a need for a biological interpretation of the results generated by the methods. There must be some processes that we should expect to see being differentially expressed between sugar rich and a sugar deprived environment. For example this may include the GO terms associated with energy metabolism, biofilm creation, growth etc. Expert curators with the knowledge of plaque bacteria could highlight few higher level GO terms that have a potential to be differentially expressed between the two conditions. If such term, selected by curators, does not exhibit metaproteome level differential expression, then we should not expect it to be picked up by any method by chance, conversely if at least one method finds such term (or its descendant) to be differentially expressed it probably won’t be by chance alone. The ranking of these curated term in the methods output could potentially highlight which of the tools is more suitable for finding biological signal in this particular metaproteome data. Of course it is important to note that the conclusions derived from this test cannot be trivially extended to all other biological datasets, nonetheless there is an insight to gain from assessing distinct aspects of the differences between the tools’ outputs.

**The reviewers have raised an important point. In this study, we have only focused minimally on biological interpretation of results - mainly since this was a comparison of a single pair of bioreactor samples (no sucrose versus sucrose) as compared to twelve pairs in the original study (Rudney *et al* 2015; PMID: 266848970). For biological interpretation of results, Rudney et al used MEGAN on twelve sample pairs. In a follow up study for metaQuantome analysis (Easterly *et al* 2019; PMID: 31235611), we reanalyzed the dataset using Unipept outputs. In the metaQuantome study we found results from the new workflow that used Unipept and metaQuantome agreed with the earlier biological outputs. In our opinion, in order to draw biological conclusions from these types of datasets it is important to carry out targeted validation of candidate peptides associated with significant functions and taxa reported in the study, and we add this point in the discussion.**

Other issues:

The authors state in the abstract that the “A BLAST analysis against the Universal Protein Knowledgebase revealed that the sensitivity and specificity of functional annotation differed between tools”. Highlighting this measure in the abstract is debatable. Primarily because there is no reason why simple BLAST against UniProt would be regarded as gold standard. If it is, there is no need for more sophisticated tools. It’s a useful measure to some extent, but it’s not clear what that measure actually conveys as it seems to me that it depends heavily on the degree of overlap between UniProt and the peptide search database (HOMD in this case). In such case almost by definition Unipept would have to generate the most precise GO, as it is directly based on the peptide matches to UniProt database. This is also what we see.

**We appreciate the reviewer’s inputs and have now used BLAST searches against NCBInr referring to this as a baseline output rather than a “gold-standard”. The BLAST2GO searches against NCBInr are now highlighted as a baseline in figures and single peptide analysis (Figures 3 and 4 and Supplementary figures S5 and S6).**

Execution time of the tools, is always an important factor in the decision which of the available tools to use. For large scale analysis it is often the deciding factor. It would be helpful to point out the total computation time of each of the pipelines that were used to generate final GO terms. If a database only starts with peptides the Mascot search should be added to the computation time, but the execution time of the tool itself should also be highlighted.

**We have provided the computational time for EggNOG mapper and Unipept 4.0 on the Galaxy interface (Supplementary S8). Since the rest of the tools were run by developers/experts on their computational infrastructure, we decided to provide details on only those tools which have been installed and run on Galaxy infrastructure.**

The authors state that DIAMOND has “similar sensitivity to BLAST”. There is a price for the speed-up of search and it seems to be sensitivity [1, Fig. 2b]. In my experience this is especially true for a very diverged proteins (identity <30%), where DIAMOND significantly loses sensitivity. The exact numbers will depend on the testing methodology.

**We have removed the statement from the manuscript.**

Figure 3. Legend should be corrected. Pink in “Type of term” legend relates to “Extraneous” terms, which are not present in the bar chart. However, in the same figure pink is used also as a color of eggNOG terms that are not part of the MetaGOmics output. This is misleading as only only one of the eggNOG terms (Formylmethionine deformylase activity”) could be considered as “extraneous” in relation to the gold terms.

**The reviewer has highlighted an important issue and has been corrected by the authors. The color scheme for Figure 3 has now changed to be focused on the types of GO relationships (ancestors, descendants). Thus, the tool colors have been omitted for clarity. Additionally, the ‘extraneous’ category has been removed.**

Reviewer #2: This is an interesting manuscript comparing different tools used for metaproteomic data processing. The manuscript has some limitations that need to be addressed.

-This seems to be a combination of comparing tools and an add-on to the Galaxy platform. Sufficient details are provided for the comparison of the tools. However, it is not possible to review the add-on to the Galaxy platform as limited details are provided. It is a bit strange to have both in one manuscript. I would suggest to focus the manuscript on comparing the tools.

**We thank the reviewers for the suggestion and have moved the figures and text to the supplementary section S7. We now instead state the importance of tool accessibility via the Galaxy framework in the discussion section.**

-Table 3. Comparing ranks is really not useful and somewhat misleading. Instead, the results from each GO term should be analyzed (# of peptide mapped and quantification) and the results statistically compared.

**We have made changes to Table 3 and state the fold-change and percentile for ranking of the GO terms. We have also provided the top five up-regulated and top five down-regulated GO terms for each tool in the supplementary section. We also provide the complete list of expand and filtered terms for molecular function, biological process and cellular component (Zenodo Link: https://zenodo.org/record/3816624).**

-Figure 4 should either be removed or moved as supplementary.

**We have moved the figures and description to the supplementary section S7, while retaining the discussion on access via Galaxy to the discussion section.**

-The authors did a good job at highlighting the limitations of their analysis.

**We appreciate the comments by the reviewer.**

-A few typos throughout the manuscripts.

**We have scanned and corrected the typos in the revised version of the manuscript.**
