# Supplementary material for: Survey of metaproteomics software tools for functional microbiome analysis
Source: PLoS One. 2020 Nov 10;15(11):e0241503. doi: 10.1371/journal.pone.0241503 (PMC7654790; doi:10.1371/journal.pone.0241503)
Supplement: Attachment — Submitted filename: Response to Reviewers PONE-D-20-04130R1.docx [file pone.0241503.s003.docx]

PONE-D-20-04130R1

Survey of metaproteomics software tools for functional microbiome analysis.

PLOS ONE

Dear Dr. Jagtap,

Thank you for submitting your manuscript to PLOS ONE. After careful consideration, we feel that it has merit but does not fully meet PLOS ONE’s publication criteria as it currently stands. Therefore, we invite you to submit a revised version of the manuscript that addresses the points raised during the review process.

Mainly, the remaining inconsistencies spotted by the reviewer should be attended to.

Please submit your revised manuscript by October 26, 2020. If you will need more time than this to complete your revisions, please reply to this message or contact the journal office at plosone@plos.org. When you're ready to submit your revision, log on to <https://www.editorialmanager.com/pone/> and select the 'Submissions Needing Revision' folder to locate your manuscript file.

Please include the following items when submitting your revised manuscript:

- A rebuttal letter that responds to each point raised by the academic editor and reviewer(s). You should upload this letter as a separate file labeled 'Response to Reviewers'.
- A marked-up copy of your manuscript that highlights changes made to the original version. You should upload this as a separate file labeled 'Revised Manuscript with Track Changes'.
- An unmarked version of your revised paper without tracked changes. You should upload this as a separate file labeled 'Manuscript'.

If you would like to make changes to your financial disclosure, please include your updated statement in your cover letter. Guidelines for resubmitting your figure files are available below the reviewer comments at the end of this letter.

If applicable, we recommend that you deposit your laboratory protocols in [protocols.io](http://protocols.io/) to enhance the reproducibility of your results. Protocols.io assigns your protocol its own identifier (DOI) so that it can be cited independently in the future. For instructions see: <http://journals.plos.org/plosone/s/submission-guidelines#loc-laboratory-protocols>

We look forward to receiving your revised manuscript.

Kind regards,

Frederique Lisacek

Academic Editor

PLOS ONE

[Note: HTML markup is below. Please do not edit.]

Reviewers' comments:

Reviewer's Responses to Questions

**Comments to the Author**

1. If the authors have adequately addressed your comments raised in a previous round of review and you feel that this manuscript is now acceptable for publication, you may indicate that here to bypass the “Comments to the Author” section, enter your conflict of interest statement in the “Confidential to Editor” section, and submit your "Accept" recommendation.

Reviewer #3: (No Response)

2. Is the manuscript technically sound, and do the data support the conclusions?

The manuscript must describe a technically sound piece of scientific research with data that supports the conclusions. Experiments must have been conducted rigorously, with appropriate controls, replication, and sample sizes. The conclusions must be drawn appropriately based on the data presented.

Reviewer #3: Yes

3. Has the statistical analysis been performed appropriately and rigorously?

Reviewer #3: Yes

4. Have the authors made all data underlying the findings in their manuscript fully available?

The [PLOS Data policy](http://www.plosone.org/static/policies.action#sharing) requires authors to make all data underlying the findings described in their manuscript fully available without restriction, with rare exception (please refer to the Data Availability Statement in the manuscript PDF file). The data should be provided as part of the manuscript or its supporting information, or deposited to a public repository. For example, in addition to summary statistics, the data points behind means, medians and variance measures should be available. If there are restrictions on publicly sharing data—e.g. participant privacy or use of data from a third party—those must be specified.

Reviewer #3: Yes

5. Is the manuscript presented in an intelligible fashion and written in standard English?

PLOS ONE does not copyedit accepted manuscripts, so the language in submitted articles must be clear, correct, and unambiguous. Any typographical or grammatical errors should be corrected at revision, so please note any specific errors here.

Reviewer #3: No

6. Review Comments to the Author

Please use the space provided to explain your answers to the questions above. You may also include additional comments for the author, including concerns about dual publication, research ethics, or publication ethics. (Please upload your review as an attachment if it exceeds 20,000 characters)

Reviewer #3: The authors describe in their manuscript a comparison of tools providing functional annotations, applied here on metaproteomics data. A preprocessed common dataset is used for all six tools. The functional annotation is investigated through directly predicted or secondarily obtained gene ontologies to have a common framework to do comparisons.

As clearly stated in the title and acknowledged by the authors, it is more a survey than a well-controlled benchmarking and it is limited to providing an overview of the available methods while demonstrating that their predictions vary substantially. The authors discuss the cause of these discrepancies as being for instance the variety of reference database, and advocate for the improvement and curation of these resources. Since the field lacks any benchmarking for that kind of aims, this study is a first step and therefore valuable.

While I appreciate the design of the different evaluations and the discussion, I find the overall description of the tools and their evaluation rather confusing, which in my opinion impair the understanding of the results if you are not a user of each of these six different pipelines. I am unsure whether this is caused by multiple errors in the manuscript or a lack of rigor in the systematic description of the inputs/outputs/databases. Different sections, figures, and tables seem to contradict or to consider the features of each tool differently. I would advocate for polishing and clarification. The language is clear.

We appreciate the comments by the reviewer. In the revised version of the manuscript we have addressed the concerns raised by making changes to the Figure 1 and text changes that offer clearer description of the methodology.

- In general, page5:"Standard procedures were used for each tool" is not sufficient to identify when methods overlap regarding their data sources. Databases underlying the tool should be listed explicitly for each of the six methods, and not scattered across different figure and tables, no matter bundled with the tool or to be provided externally.

We have made changes to Figure 1 that has a “Database(s)” section that outlines which internal databases were used by each software tool. We hope that sets the stage for the reader to navigate the manuscript.

- For instance, eggNOG db is used by multiple tools, probably for different purpose. Figure 1 does not connect eggNOG db to MEGAN, while methods state page5:"the analysis was performed using eggNOG ". Table 2 states that eggNOG obtain functional annotation of type "proteins" while MEGAN obtain them from eggNOG groups. All together, this prevents the reader from understanding whether MEGAN and eggNOG get part of their functional annotation from a common source or not at all.

We thank the reviewer for pointing this out. The new “Database(s)” section in the revised Figure 1 now explicitly outlines which databases are used for each software tool, with particular emphasis on EggNOG and UniProtKB databases. In the updated Figure 1, MEGAN is now connected to the eggNOG database.

Additionally, Table 1 (which the reviewer has referred to as “Table 2”) is revised from stating “proteins” to “eggNOG orthologous groups” as an annotation type, to demonstrate similarity with MEGAN.

- Figure 1 is not that helpful as it does not reflect the difference between tools that matter during the evaluation. Especially, it does not clearly separate and define the databases used for classification of the input and their functional annotation. In some part of the manuscript, other db (page5: "For MetaGOmics, a list of peptides and the HOMD were uploaded (...)") are mentioned but not present on Figure 1.

The issue is now addressed via the new “Database(s)” section in Figure 1. We have also added an input element - Human Oral Microbiome Database (HOMD) - that connects to MetaGOmics software so that there is consistency between the figure and description.

- Two tools are described as natively predicting GO, vs four which do not (page8:"tools that natively output GO terms (i.e., Unipept and MetaGOmics)"). But on Figure 1, five out of six tools output GO, so this seems to be a different interpretation of natively predicting GO. Moreover, methods states that page6:"GO terms (...) are common annotation type throughout most of the tools (i.e., eggNOG-mapper and Unipept) (...) However, MEGAN, ProPHAnE, and MPA do not provide direct GO term outputs". Only five tools mentioned, most of the tools being two of them (!), and not matching previous statements or Figure1.

We have addressed this by removing the misleading “GO terms” row in the table in Figure 1. Now, the revised figure, via the “Output” section, clearly illustrates native GO term outputs from Unipept and MetaGOmics.

- I am unsure whether MPA uses the output of XTandem and Peptideshaker since the "input" folder in the supplementary archive contain a fasta that contains cRAP protein, which resemble a database use for search and filtering (277K_for_737_MPA_with_cRAP.fasta). I suppose this could indicate the actual input is the initial spectrum files identical to what is given to XTandem (in methods: page5:"For MPA, the same parameters as for SearchGUI were used"). This would contradict the statement in the discussion page21:"While using the same post-search inputs from the same dataset". Or there is something else missing (cRAP-filtereds spectrum files?) as input for MPA in the supplementary archive.

The reviewer raises important questions on how MPA analysis was carried out. This was amended by specifying that MPA performs its own searches, using the same inputs (MGF files) and parameters as SearchGUI and requires a FASTA database that was generated using a database sectioning approach. The statement in the discussion (page 21) referred to by the reviewer was modified to convey this information.

- To clarify all the points above, a visual summary of the workflow from the first difference appearing in any tool to the end of the process with the translation of GO would be beneficial, either as supplementary or as replacement of Figure 1.

Figure 1 has now been revised to illustrate differences and similarities between tools more easily via a grid-like and color-coded design. Additionally, a dedicated section for “GO Term translation” is included within Figure 1.

- The definition of peptide-centric / peptide-level / protein-centric / protein-level could be clarified, if these terms are necessary to understand the input and outputs that lead to GO predictions attached to individual proteins, OG, or peptides. These terms seem to be used in contradictory ways and are confusing.

page9: "Of the tools that used peptide-level information (see Figure 1)". => Figure 1 contains "protein-level information" as possible output, and "peptides" as possible input. The reader could assume that "peptide-level information" are those without the checkmark for "protein-level information" outputs in Figure 1, but "used" does not mean "outputed".

This is addressed by including the word “input” in the sentence to clarify that peptide-level inputs were used for these tools. “Input” was used rather than “output” due to the clear peptide-level input information in Figure 1.

Page9: "tools that provided peptide-level outputs were used, thus excluding MEGAN, MPA, and ProPHAnE" This does not match any of the possibilities depicted in Figure 1, but does match page10:"analysis level" in the results section.

This is addressed by removing the “Protein level information” row in the table in Figure 1. The wording was also adjusted to center around “peptide inputs”.

page17: " protein-level tools is highlighted here (MEGAN and ProPHAnE) " Again, this is not the same list... the meaning might be "some of the protein-level tools" but the general confusion about these terms cast doubts.

The language here is changed to look at tools that “output orthologous groups”

page18: "a peptide-centric (eggNOG-mapper, MEGAN, MetaGOmics and Unipept) or protein-centric approach (MPA and ProPHAnE)." This matches the grouping in figure1 and seems correct, but "centric" does not tell whether to focus on inputs or outputs and MPA and ProPHAnE do not have the same input type according to figure 1.

Instead of using “peptide-centric” and “protein-centric”, the sentence was modified to divide the tools between those that use “peptide-level inputs” and those that use “other means”.

- page16: "The single- peptide analysis (Figure 3, Supplement S5 and Supplement S6) was carried out for Unipept, MetaGOmics and eggNOG-mapper since they provide GO term annotations at a peptide level. As a result, we do not have results for the protein-level tools MPA and ProPHAnE." Related to previous comments, MEGAN is not included, no justification here... is it because of protein-level vs peptide-level? Or inhability to provide GO term ?

MEGAN was not initially included, due to mislabeling on the previous Figure 1. Now, it has been added (and Figure 1 has been corrected).

minor:

- the Jaccard Index is only mentioned in the discussion while it was removed elsewhere during refactoring. Please check whether it is still intended to refer to this or prefer something else to match figure 2

The “Jaccard Index” was now correctly renamed to “overlap index”

- MEGAN5 and MEGAN6 are mentioned as being the evaluated version. Please check whether this is a typo.

This is a typo. MEGAN5 was used for this survey.

- Supplementary figure S5: one color is not labeled (pink)

Color has been added (pink) for the “Extraneous” category.

7. PLOS authors have the option to publish the peer review history of their article ([what does this mean?](https://journals.plos.org/plosone/s/editorial-and-peer-review-process#loc-peer-review-history)). If published, this will include your full peer review and any attached files.

If you choose “no”, your identity will remain anonymous but your review may still be made public.

**Do you want your identity to be public for this peer review?** For information about this choice, including consent withdrawal, please see our [Privacy Policy](https://www.plos.org/privacy-policy).

Reviewer #3: No
